# Causal Mediation Analyses for the Natural Course of Hepatitis C: A Prospective Cohort Study

**DOI:** 10.2188/jea.JE20240034

**Published:** 2025-01-05

**Authors:** Yi-Ting Huang, Yao-Chun Hsu, Hwai-I Yang, Mei-Hsuan Lee, Tai-Hsuan Lai, Chien-Jen Chen, Yen-Tsung Huang

**Affiliations:** 1 Institute of Statistical Science, Academia Sinica, Taipei, Taiwan; 2 Institute of Epidemiology and Preventive Medicine, National Taiwan University, Taipei, Taiwan; 3 Center for Liver Diseases, E-Da Hospital, Kaohsiung, Taiwan; 4 School of Medicine, I-Shou University, Kaohsiung, Taiwan; 5 Department of Medicine, Fu-Jen Catholic University Hospital, New Taipei, Taiwan; 6 Institute of Biomedical Informatics, Yangming Campus, National Yang Ming Chiao Tung University, Taipei, Taiwan; 7 Genomics Research Center, Academia Sinica, Taipei, Taiwan; 8 Institute of Clinical Medicine, Yangming Campus, National Yang Ming Chiao Tung University, Taipei, Taiwan; 9 Graduate Institute of Medicine, Kaohsiung Medical University, Kaohsiung, Taiwan; 10 Biomedical Translation Research Center, Academia Sinica, Taipei, Taiwan; 11 Division of Nephrology, Department of Internal Medicine, National Taiwan University Hospital, Taipei, Taiwan; 12 Department of Mathematics, National Taiwan University, Taipei, Taiwan; 13 Department of Applied Mathematics, National Sun Yat-sen University, Kaohsiung, Taiwan

**Keywords:** cohort study, disease burden, mediation analysis, natural history, hepatitis C

## Abstract

**Background:**

Hepatitis C virus (HCV) infection is a systemic disease. However, the relative contribution of intrahepatic and extrahepatic diseases to mediating HCV-induced mortality is unclear, albeit critical in resource allocation for reducing preventable deaths. To this end, this study comprehensively quantified the extent to which intrahepatic and extrahepatic diseases mediate HCV-induced mortality.

**Methods:**

A community-based cohort study with >25 years of follow-up was conducted in Taiwan. HCV infection was profiled by antibodies against HCV and HCV RNA in participants’ serum samples. The cohort data were linked to Taiwan’s National Health Insurance Research Database to determine the incidences of potential mediating diseases and mortality. We employed causal mediation analyses to estimate the mediation effects of HCV on mortality in relation to the incidences of 34 candidate diseases.

**Results:**

In 18,972 participants with 934 HCV infection, we observed that 54.1% of HCV-induced mortality was mediated by intrahepatic diseases, such as liver cirrhosis and liver cancer, and 45.9% of mortality was mediated by extrahepatic diseases. The major extrahepatic mediating diseases included septicemia (estimated proportion of HCV-induced mortality mediated through the disease: 25.2%), renal disease (16.7%), blood/immune diseases (12.2%), gallbladder diseases (9.7%), and endocrine diseases (9.6%). In women, hypertension (20.0%), metabolic syndrome (18.9%), and type 2 diabetes (17.0%) also mediated HCV-induced mortality. A dose-response relationship of HCV viral load was further demonstrated for the mediation effect.

**Conclusion:**

Both intrahepatic and extrahepatic manifestations mediated approximately half of HCV-induced mortality. The mediation mechanisms are supported by a dose-response relationship of HCV viral load.

## INTRODUCTION

Chronic hepatitis C virus (HCV) infection increases the incidences of systemic diseases and mortality.^1^ Direct-acting antiviral agents can result in a sustained virological response and even clear HCV infection^2^; however, their accessibility remains limited in most HCV-endemic areas.^3^ HCV vaccines with documented efficacy and safety in humans have yet to be developed.^4^ Although the World Health Organization aims to eliminate HCV by 2030, approximately 58 million HCV carriers, with 1.5 million new cases reported per year worldwide; more than 399,000 individuals died of HCV infection in 2019.^5^ The disease burden of HCV infection, in terms of morbidity and mortality, is a crucial issue in clinical medicine and public health. Hence, understanding the role of HCV-induced diseases in HCV-related mortality can guide health policies in screening particular diseases (except liver diseases) to reduce the preventable deaths of the HCV-infected population.

This study examined the natural course of HCV and identified diseases linking HCV infection and mortality. HCV may cause mortality through intrahepatic or extrahepatic diseases. Liver cancer is the major cause of death in HCV carriers, and the infection progresses from repeated necroinflammation, fibrosis/cirrhosis, to liver cancer and eventually to death.^6^ Extrahepatic manifestations of HCV infection have also been documented by the association of HCV with the incidences of non-hepatic diseases^1^^,^^7^^–^^9^ and mortality caused by non-hepatic diseases.^10^^–^^12^ However, no studies have evaluated the role of diseases in mediating the mechanism of HCV-induced mortality, which is critical in the allocation of resources for preventing HCV-related mortality. Moreover, studies may have underestimated the importance of extrahepatic diseases. In particular, HCV-associated extrahepatic diseases and liver-related mortality exemplify semi-competing risks^13^ wherein patients who die rapidly from liver diseases do not have the opportunity to develop extrahepatic disorders, whereas those with chronic extrahepatic disorders still have a risk of mortality. However, studies on disease- or cause-specific mortality have not focused on this issue. Therefore, the contribution of extrahepatic disorders to mortality may be underestimated or even mistakenly considered protective.

To more effectively investigate the roles of intrahepatic and extrahepatic disorders in HCV-induced mortality, we employed a mediation model separating disease-specific mortality into disease incidence and mortality, which served as a mediator and an outcome in the proposed model, respectively. The mediation model was proposed to investigate the effect among an exposure, a mediator, and an outcome,^14^ with the mediation effect representing the effect of the exposure on the outcome through the mediator and the alternative effect representing the effect of the exposure on the outcome not mediated through the mediator.^14^ A series of causal mediation analyses for various types of mediators or outcomes have been developed^14^ and applied in biomedical studies to investigate disease progression and vaccine efficacy.^15^^,^^16^ We adopted a causal mediation model that was developed for the setting in which the mediator and outcome were both time-to-event.^13^

Although HCV infection has been associated with a variety of diseases and mortality, how these diseases mediate the causal effect of HCV infection on mortality remains unclear. In this study, we examined the natural course of HCV infection by investigating the role of intrahepatic and extrahepatic manifestations.

## METHODS

### Study population

We recruited a community-based cohort in Taiwan from the Risk Evaluation of Viral Load Elevation and Associated Liver (REVEAL) study conducted between 1991 and 1992.^17^ Blood samples were obtained from recruited participants, and their serum samples were divided for screening viral hepatitis infection, biochemistry tests, and hematology examinations. Researchers conducted a personal interview and assisted each participant in completing the structured health assessment questionnaire. At the end of 1992, 23,820 residents were recruited. The cohort was followed prospectively and linked to the National Health Insurance Research Database (NHIRD), a nationwide population-based database deriving from the claims data of beneficiaries enrolled in the National Health Insurance, to update their health conditions until the end of 2017.

### Definitions of HCV infection, potential mediating diseases, mortality, and main factors

Chronic HCV and hepatitis B virus (HBV) infections as binary exposures were characterized using antibodies against HCV (anti-HCV) and hepatitis B surface antigen (HBsAg), respectively, in serum samples at cohort entry. We excluded HBsAg-positive participants. The viral load of the anti-HCV-positive participants was examined by measuring HCV RNA in serum samples. To identify the participants’ health status, REVEAL data were linked to the NHIRD to determine the potential mediating diseases and mortality. We preselected 97 categories of potential mediating diseases from different organ systems on the basis of a comprehensive literature review.^1^^,^^7^^–^^12^ Diseases or manifestations as time-to-event mediators were ascertained using inpatient and outpatient records, prescription records, the National Cancer Registry, or the Catastrophic Illness Data of the NHIRD; details are provided in eMaterial 1. Intrahepatic manifestations included liver fibrosis, cirrhosis, liver cancer, and cholangiocarcinoma. For the 97 preselected diseases, we excluded those with a prevalence of <10% or >90%, considering the lack of statistical power when dealing with outcomes that are too rare or common. Finally, 34 potential mediating diseases were included in the subsequent statistical analyses (eTable 1). All-cause mortality and disease-specific mortality as time-to-event outcomes were ascertained by linking to the Cause of Death data in the NHIRD. HCV-infected participants who were prescribed medication for HCV infection were censored at the time of prescription (*n* = 130). Other approaches of handling the treatment were included in the sensitivity analysis in eMaterial 2.

### Statistical analysis

Characteristics at cohort entry, namely age (categorized into 30–39, 40–49, 50–59, and ≥60 years), sex, alcohol consumption, cigarette smoking, and alanine aminotransferase (ALT) levels (<15, 15–44, and ≥45 IU/L), are presented as the number of each category with the percentage. Fisher’s exact test was used to examine the association between categorical characteristics and chronic HCV infection. Baseline covariates and their two-way interactions were adjusted in statistical analyses. We conducted conventional analyses to evaluate the association of chronic HCV infection with (1) incidences of potential mediating diseases and (2) all-cause mortality and disease-specific mortality. Crude incidence rates were calculated, and the Cox proportional hazards model was used to estimate adjusted hazard ratios while controlling for the aforementioned covariates. Moreover, analyses were conducted by stratifying HCV RNA viral load into undetectable (<25 IU/mL) and detectable (≥25 IU/mL).

#### Causal mediation analysis

**Figure 1.**
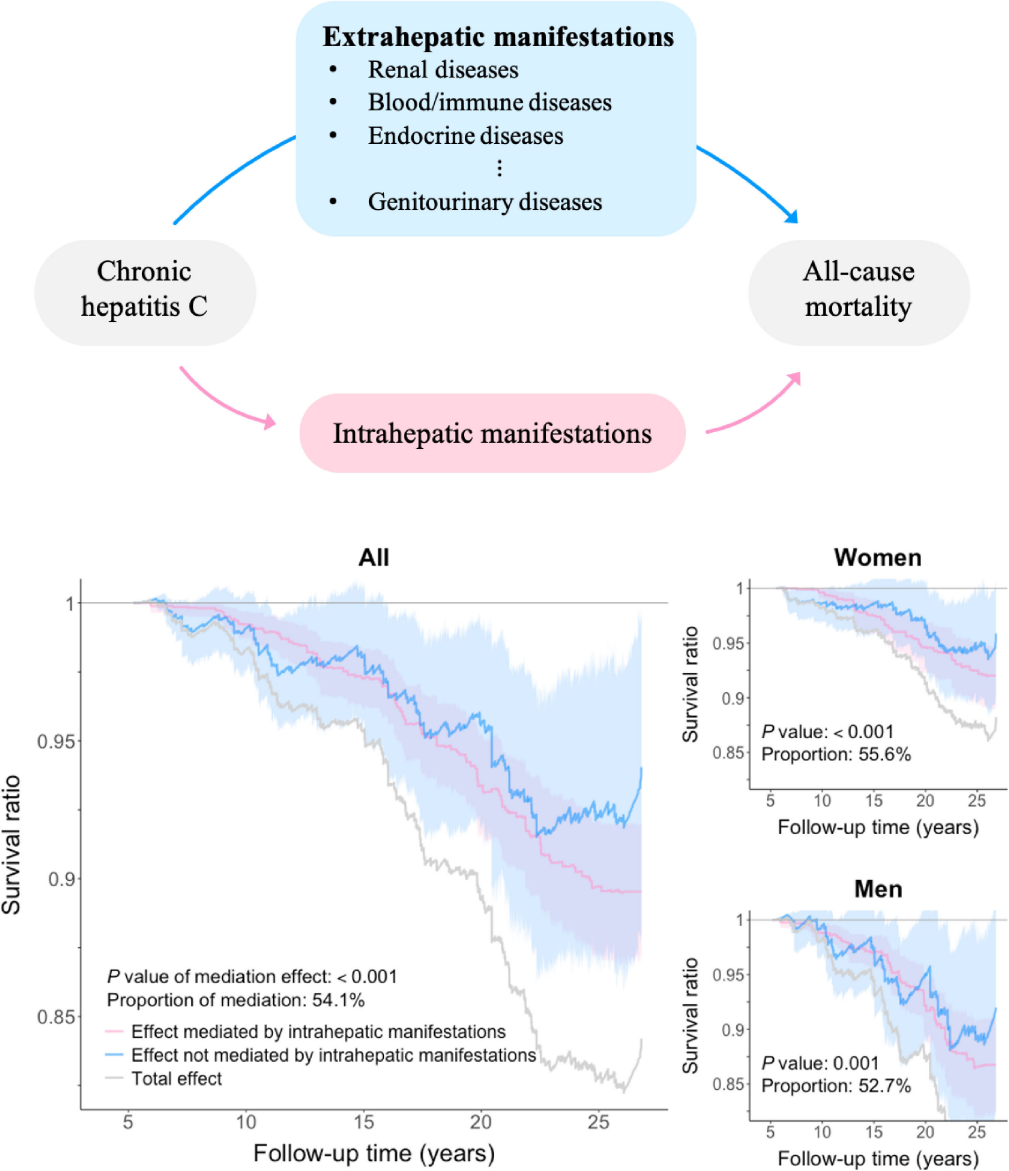
Survival ratio of hepatitis C virus on mortality mediated by intrahepatic manifestations. The survival ratio was estimated using the nonparametric causal mediation estimator with inverse probability weighting (IPW). In the total population, IPW was implemented with the probability of hepatitis C virus infection estimated from logistic regression on the following variables: age group (30–39 [reference], 40–49, 50–59, and 60–65 years), sex (reference: women), alcohol consumption (reference: yes), cigarette smoking (reference: yes), alanine transaminase (ALT) levels (<15 (reference), 15–44, and ≥45 IU/L), interaction between the age group (40–49 years) and sex, interaction between the age group (40–49 years) and ALT levels (15–44 IU/L), interaction between sex and alcohol consumption, interaction between sex and ALT levels (≥45 IU/L), interaction between cigarette smoking and alcohol consumption, and interaction between cigarette smoking and ALT levels (15–44 IU/L). In sex-specific analysis, similar IPW was implemented except for sex and its associated interactions.

In our study, we have enumerated all possible mechanisms from hepatitis C to mortality. If the intrahepatic manifestations are construed as the mediator, the path through the extrahepatic manifestations is the direct effect, and vice versa (upper panel in Figure 1). To identify the mediating diseases of HCV-induced mortality, we conducted a series of mediation analyses. We first performed hypothesis testing for the 34 potential mediating diseases one at a time by using the joint significance test.^13^ The *q* value was calculated to control for the false discovery rate in multiple comparisons. For significant mediating diseases, we performed nonparametric causal mediation analysis^13^ to estimate the mediation and alternative effects of HCV infection on mortality in relation to intrahepatic and extrahepatic diseases. The mediation analysis would be conducted individually for each potential mediating disease to assess the overall impact of HCV carrier status through that specific mediating disease on mortality. Additionally, we calculated the proportion of mediation (PM) by determining the ratio of the mediation effect to the total effect across the study period, which quantified the extent to which HCV-induced mortality was mediated by the disease. The 95% confidence intervals (CIs) of the effects were computed through bootstrapping by resampling the data 1,000 times. The details of the mediation analysis are provided in eMaterial 2.

We included age groups, sex, alcohol consumption, cigarette smoking, ALT levels, and their two-way interactions as confounders based on clinical significance and selected them by employing a stepwise method. Moreover, we adopted inverse probability weighting (IPW) to adjust for confounding factors and modeled the probability of HCV infection. Subsequently, we reweighted HCV-positive participants by the inverse of the probability of HCV infection and HCV-negative participants by the inverse of the probability of no HCV infection. Furthermore, we multiplied the proportion of HCV-positive or HCV-negative patients to construct stabilized weights.

## RESULTS

**Figure 2.**
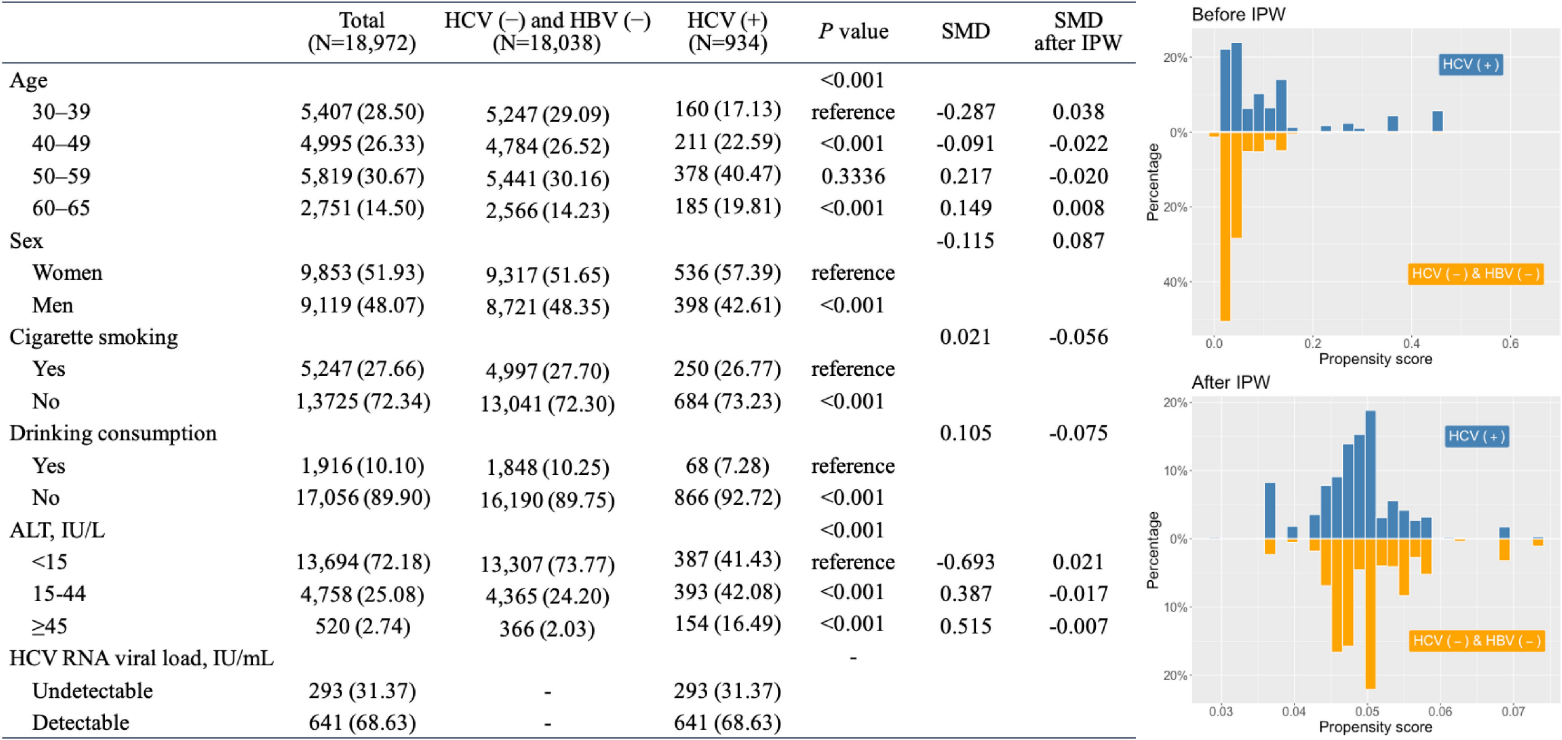
Characteristics of the REVEAL cohort by chronic hepatitis C status. Descriptive statistics are presented as the number (percentage). *P* values were calculated using Fisher’s exact test. The IPW was estimated using a logistic regression model after adjustment for age groups (30–39 [reference], 40–49, 50–59, and 60–65 years), sex (reference: women), alcohol consumption (reference: yes), cigarette smoking (reference: yes), ALT levels (<15 IU/L [reference], 15–44 and ≥45 IU/L), interaction between the age group (40–49 years) and sex, interaction between the age group (40–49 years) and ALT levels (15–44 IU/L), interaction between sex and alcohol consumption, interaction between sex and ALT levels (≥45 IU/L), interaction between cigarette smoking and alcohol consumption, and interaction between cigarette smoking and ALT levels (15–44 IU/L). ALT, alanine transaminase; HBV, hepatitis B virus; HCV, hepatitis C virus; IPW, inverse probability weighting; SMD, standardized mean difference.

This study included 18,972 participants without HBV, of whom 934 were HCV carriers and 18,038 were non-HCV carriers (Figure 2). Generally, a higher proportion of participants who were positive for anti-HCV were older, were women, and did not consume alcohol or smoke but had higher ALT levels. The distribution of propensity scores for having HCV infection was skewed to the right in participants who were positive for anti-HCV (upper right panel of Figure 2) but became similar to that in participants who were negative for anti-HCV after IPW (lower right panel of Figure 2). The balance of covariates was also found by standardized mean differences (Figure 2) and *P*-values (eTable 2).

We investigated the effect of HCV infection on incidences of the 34 potential mediating diseases (eTable 3). Increased adjusted hazard ratios of HCV infection were observed for almost all potential mediating diseases. A significant dose-response relationship with HCV viral load was observed for lupoid hepatitis, other liver disorders, and fibrosis/cirrhosis of the liver. In addition to disease incidence, HCV significantly affected mortality (all causes or causes excluding some intrahepatic disorders) with consistent dose-response relationships. We observed that the effect of HCV on disease incidence, albeit informative, was subject to the semi-competing risk of mortality.

**Figure 3.**
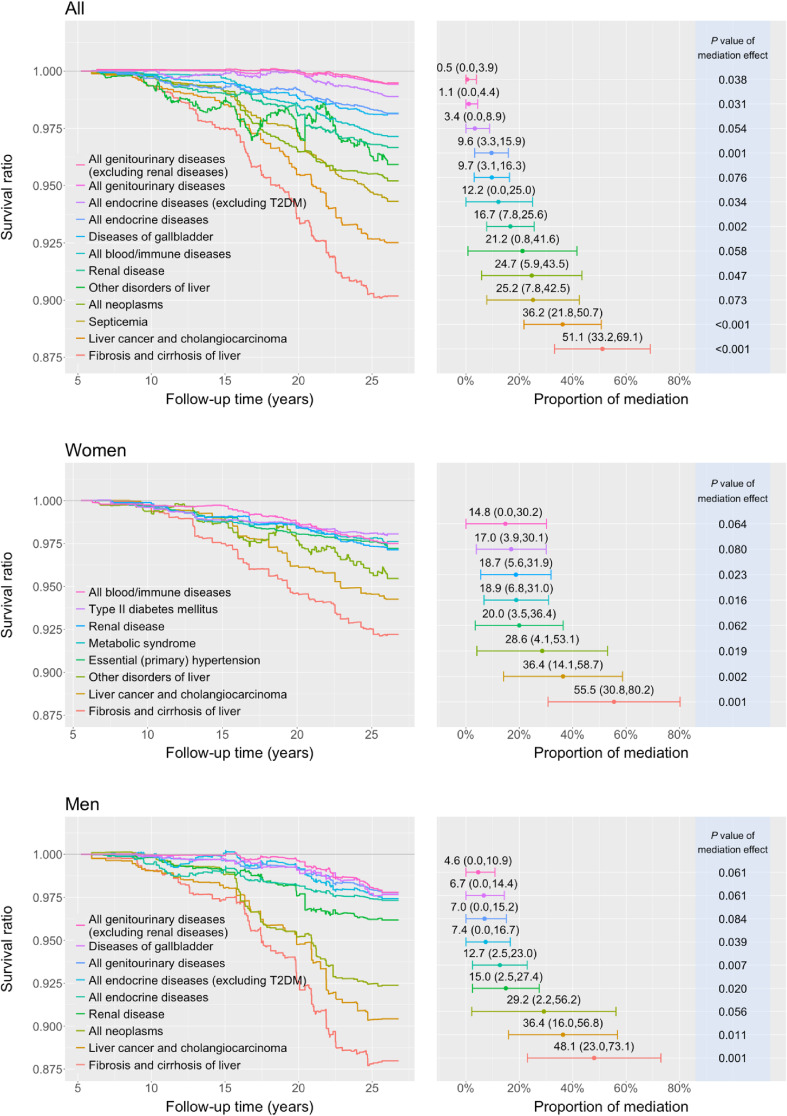
Hepatitis C virus-induced mortality by different mediating diseases. Inverse probability weighting adjustment was the same as that in Figure 2. The proportion of mediation was determined by computing the cumulative hazard difference and calculated as the mediation effect divided by the total effect. Confidence intervals of the mediation proportion were truncated at 0 if they were <0. Other disorders of the liver: excluding fibrosis and cirrhosis of liver, and fatty liver; renal disease: nephritis, nephrotic syndrome, and nephrosis.

The results of the causal mediation analysis with intrahepatic manifestations as a mediator indicated that the effect of HCV infection on mortality was significantly mediated by intrahepatic manifestations in the entire population or subgroups (female and male subgroups; Figure 1). The PMs in the entire population, women, and men were 54.1%, 55.6%, and 52.7%, respectively, suggesting that extrahepatic disorders caused almost half of the HCV-induced mortality (blue curves in Figure 1). We conducted hypothesis testing for the 34 preselected potential mediating diseases for the total, female, and male populations (eTable 4). In the total population, 12 mediating diseases had a *q* value of <0.1 (Figure 3). Significant extrahepatic manifestations included diseases from different organ systems, such as blood/immune, digestive, endocrine, renal, and genitourinary systems.

To compare our proposed effect on disease-mediating mortality with the effect on disease-specific mortality in conventional analyses (using cumulative hazard ratio [CHR] in Cox models), we expressed the mediation effect as the 25-year CHR (Table 1). The total effects (the row of all-cause mortality in Table 1) were similar between the two analyses. For disease-specific mortality, HCV effects were exaggerated in intrahepatic diseases such as liver cirrhosis (CHR 14.03; 95% CI, 6.26–31.41) and liver cancer (CHR 11.81; 95% CI, 8.53–16.35); these may bias the effects of extrahepatic diseases toward the null or even protective (eg, endocrine diseases excluding type II diabetes mellitus [T2DM]: CHR 0.75; 95% CI, 0.36–1.60). By contrast, disease-mediating mortality provided a comparable scale to examine the role of various diseases in the mechanisms underlying HCV-induced mortality: liver cirrhosis, CHR 1.31 (95% CI, 1.22–1.41); liver cancer, CHR 1.21 (95% CI, 1.15–1.29); and endocrine diseases excluding T2DM, CHR 1.02 (95% CI, 1.00–1.05).

**Table 1.** Hazard ratios of hepatitis C for disease-specific mortality or disease-mediating mortality

Significant mediating disease	Disease-specific mortality	Disease-mediating mortality

	Cox model CHR (95% CI)	Mediation model 25-year CHR (95% CI)
All		

All-cause mortality	1.74 (1.51–2.02)	1.78 (1.51–2.05)
Septicemia	0.80 (0.26–2.47)	1.14 (1.04–1.26)
All blood/immune diseases	—	1.07 (1.01–1.13)
All endocrine diseases	1.27 (0.77–2.09)	1.04 (1.02–1.07)
All endocrine diseases (excluding T2DM)	0.75 (0.36–1.60)	1.02 (1.00–1.05)
Fibrosis and cirrhosis of liver	14.03 (6.26–31.41)	1.31 (1.22–1.41)
Other disorders of liver	3.73 (1.59–8.71)	1.10 (1.00–1.20)
Diseases of gallbladder	—	1.05 (1.01–1.08)
All genitourinary diseases	1.99 (1.16–3.41)	1.01 (1.00–1.02)
All genitourinary diseases (excluding renal diseases)	2.27 (1.21–4.27)	1.01 (0.99–1.02)
Renal disease	1.31 (0.52–3.31)	1.08 (1.04–1.13)
All neoplasms	2.53 (2.05–3.12)	1.12 (1.02–1.22)
Liver cancer and cholangiocarcinoma	11.81 (8.53–16.35)	1.21 (1.15–1.29)

Women		

All-cause mortality	1.77 (1.44–2.16)	1.81 (1.46–2.18)
All blood/immune diseases	—	1.08 (1.01–1.16)
Type II diabetes mellitus	1.46 (0.59–3.59)	1.07 (1.01–1.13)
Metabolic syndrome	—	1.08 (1.02–1.14)
Essential (primary) hypertension	—	1.09 (1.01–1.18)
Fibrosis and cirrhosis of liver	7.49 (1.85–30.24)	1.34 (1.21–1.50)
Other disorders of liver	6.20 (2.03–18.90)	1.16 (1.04–1.32)
Renal disease	1.37 (0.39–4.76)	1.10 (1.04–1.16)
Liver cancer and cholangiocarcinoma	11.52 (7.08–18.72)	1.22 (1.13–1.34)

Men		

All-cause mortality	1.74 (1.43–2.12)	1.75 (1.40–2.07)
All endocrine diseases	1.18 (0.53–2.67)	1.05 (1.01–1.09)
All endocrine diseases (excluding T2DM)	0.28 (0.04–2.04)	1.04 (1.00–1.08)
Fibrosis and cirrhosis of liver	20.95 (8.08–54.36)	1.29 (1.18–1.46)
Diseases of gallbladder	—	1.04 (1.00–1.08)
All genitourinary diseases	1.59 (0.67–3.78)	1.04 (0.99–1.08)
All genitourinary diseases (excluding renal diseases)	1.76 (0.58–5.33)	1.04 (0.99–1.07)
Renal disease	1.31 (0.36–4.76)	1.07 (1.01–1.15)
All neoplasms	2.62 (1.99–3.47)	1.15 (1.00–1.29)
Liver cancer and cholangiocarcinoma	11.87 (7.79–18.10)	1.21 (1.12–1.34)

CHR, cumulative hazard ratio; CI, confidence interval; T2DM, type 2 diabetes mellitus.

The symbol “—” indicates that the model estimation did not converge.

The hazard ratios for disease-specific mortality were estimated using the Cox proportional hazards model with inverse probability weighting (IPW) adjustment for the propensity of hepatitis C virus infection.

The cumulative hazard ratios for disease-mediating mortality were estimated using the nonparametric causal mediation estimator with IPW adjustment.

Other disorders of the liver: excluding fibrosis and cirrhosis of liver, and fatty liver; renal disease: nephritis, nephrotic syndrome, and nephrosis.

The relative contribution of each mediating disease to HCV-induced mortality during the 27 years of follow-up is illustrated in Figure 3. The majority of mediating diseases exhibited an increased mediation risk over time. The two most crucial mediating diseases were fibrosis/cirrhosis of the liver and liver cancer (including cholangiocarcinoma), with PMs of 51.1% and 36.2%, respectively, in total population (55.5% and 36.4% in women and 48.1% and 36.4% in men, respectively). In the descending order of PM, extrahepatic mediating diseases included septicemia, renal disease (nephritis, nephrotic syndrome, and nephrosis), blood/immune diseases, gallbladder diseases, endocrine diseases, endocrine diseases excluding T2DM, genitourinary diseases, and genitourinary diseases excluding renal disease, with the respective PM of 25.2%, 16.7% (18.7% in women; 15.0% in men), 12.2%, 9.7% (6.7% in men), 9.6% (12.7% in men), 3.4% (7.4% in men), 1.1% (7.0% in men), 0.5% (4.6% in men). Moreover, hypertension (PM: 20.0%), metabolic syndrome (18.9%), and T2DM (17.0%) were mediating diseases in women.

**Figure 4.**
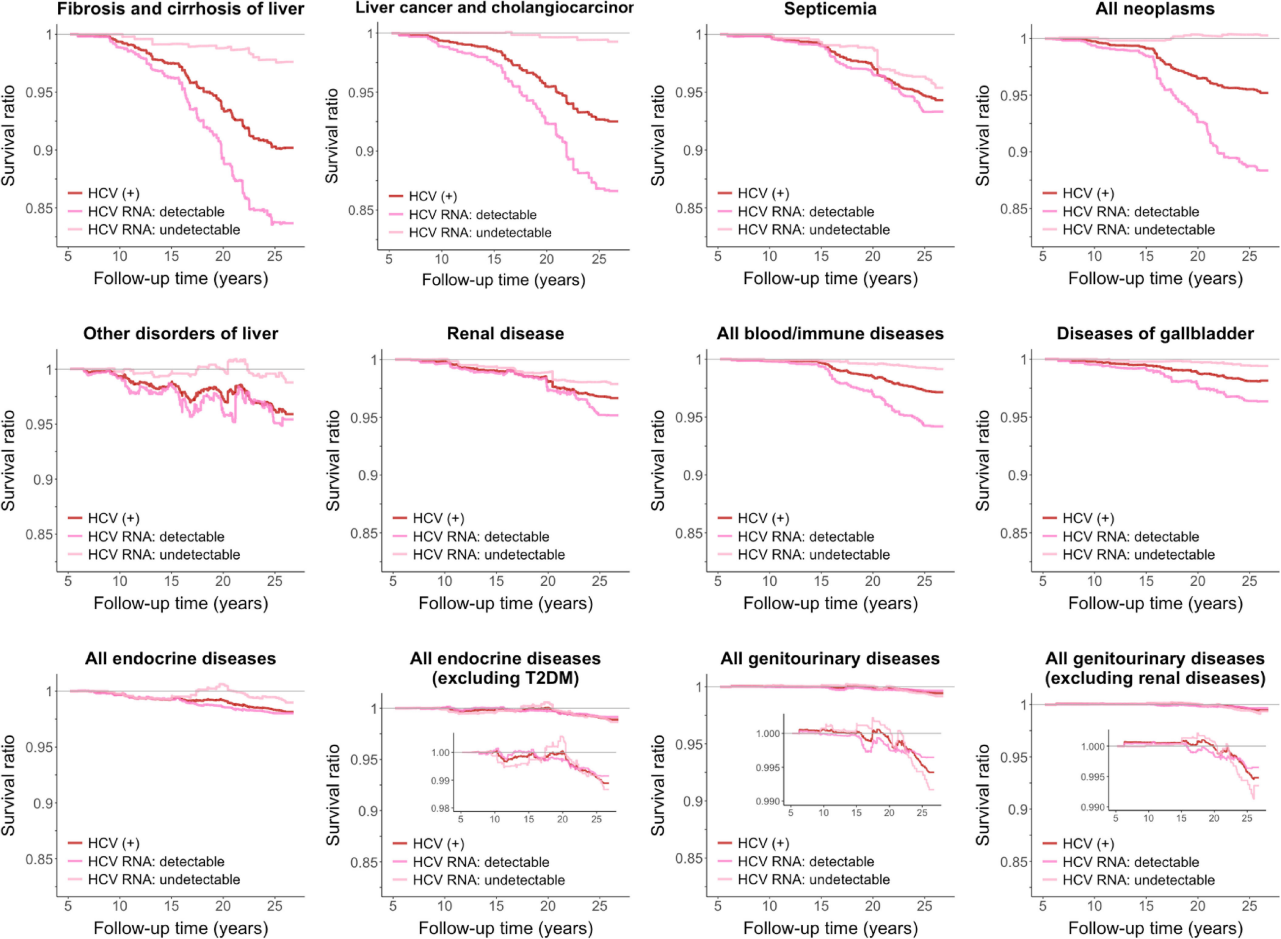
Survival ratio of disease-mediating HCV-induced mortality stratified by HCV viral load. The survival ratio was estimated using the nonparametric causal mediation estimator with inverse probability weighting adjustment for the age group (30–39 [reference], 40–49, 50–59, and 60–65 years), sex (reference: women), alcohol consumption (reference: yes), cigarette smoking (reference: yes), alanine transaminase (ALT) levels (<15 [reference], 15–44, and ≥45 IU/L), interaction between the age group (40–49 years) and sex, interaction between the age group (40–49 years) and ALT levels (15–44 IU/L), interaction between sex and alcohol consumption, interaction between sex and ALT levels (≥45 IU/L), interaction between cigarette smoking and alcohol consumption, and interaction between cigarette smoking and ALT levels (15–44 IU/L).

By stratifying the participants who were positive for anti-HCV into detectable and undetectable HCV RNA subgroups, we demonstrated a dose-response relationship of the mediation effects for the identified mediating diseases in the total (Figure 4), female (eFigure 2), and male (eFigure 3) populations. The identified mediation effects were robust even in the presence of unmeasured confounding and selection bias by treatment censoring, as indicated in sensitivity analyses (eFigure 4, eFigure 5, eFigure 6, and eTable 5), with details provided in eMaterial 2.

## DISCUSSION

The study aims to investigate how chronic HCV infection raised the risk of mortality rate through different diseases using a representative cohort in Taiwan. Our analyses show that statistically significant mediating diseases are not only the intrahepatic disorders (PM: 54.1%) but also the extrahepatic manifestations 45.9%), including septicemia, renal disease, blood/immune diseases, gallbladder diseases, endocrine diseases, endocrine excluding T2DM, and genitourinary diseases. The validity of the findings is supported by the dose-response relationship of HCV viral load as well as several sensitivity analyses. The results not only help understand the natural course of hepatitis C infection but also have public health implications.

### Mechanism linking HCV infection with extrahepatic diseases

One of the mechanisms of HCV-induced extrahepatic manifestations involves microRNA communication between hepatocytes and the immune system.^18^ Expression of B-cell activating factor is upregulated by GU (guanine-uracil)-enriched microRNAs through exosome transmission and the Toll-like receptor 7 activation.^18^ Thus, the immune system is subverted, resulting in the subsequent induction of autoimmunity by HCV infection.^19^^,^^20^ HCV drives autoimmune alterations or inflammatory cytokines to promote the development of circulatory system diseases and enhances the expression of antiendothelial antibodies, thus generating oxidative stress and insulin resistance.^21^^,^^22^ Immune- and metabolism-related pathways may be involved in the development of HCV-induced endocrine diseases. HCV-infected hepatocytes may upregulate lipid and glucose metabolism.^23^ Moreover, HCV interferes with the insulin signaling pathway by upregulating the inflammatory cytokine tumor necrosis factor-α, hypophosphorylating insulin receptor substrate-1 and -2, phosphorylating protein kinases, upregulating gluconeogenic genes, accumulating lipids, and targeting lipid storage organelles.^24^ HCV-induced renal diseases may result in cryoglobulinemia, viral antigen–antibody complexes, or a direct viral cytopathic effect.^25^ Endothelial damage in renal disorders is caused by the pathway of complement activation that generates chemotactic factors and then activates proinflammatory leucocytes.^25^ In addition, direct viral invasion of the renal parenchyma may be the leading cause of renal disease.^25^ An observational study has shown improved renal outcomes after antiviral treatment for HCV infection.^26^ In gallbladder disease, HCV alters the bile composition that facilitates the formation of gallbladder stones.^27^ Furthermore, HCV RNA was found in gallbladder cell culture, and HCV core antigens can be detected in the proliferated bile duct epithelium.^27^

### Competing risks

Although disease-specific mortality focuses on both disease and death as a composite outcome, our proposed disease-mediating mortality separates them into a mediator (disease) and an outcome (mortality) in the natural course of HCV (Figure 1). Using the mediation model to characterize the natural course, we quantified the relative importance of each individual disease in the mechanism of HCV-induced mortality. Cause- or disease-specific mortality has been widely used in clinical and epidemiological studies as an outcome to examine the effect of exposure on specific diseases. Such effect measures may avoid the underestimated incidences of rapidly progressing diseases. However, disease-specific mortality is still subject to competing risks; for example, an increase in liver disease-related mortality by HCV may implicitly prevent non-liver-related mortality (Table 1). Moreover, certification for a cause of death is subject to systematic bias and uncertain accuracy.^28^

To examine diseases that mediate the natural course from HCV infection to mortality, the association of HCV with the incidences of various diseases can be estimated. Such analyses are subject to semi-competing risks by death^13^; for example, HCV carriers who die from other causes do not have the opportunity to develop renal diseases, but those who develop renal diseases still have a risk of mortality. In HCV carriers, the course of dying from intrahepatic diseases, such as HCC and liver cirrhosis, is faster than that from extrahepatic diseases. The effect estimates of HCV on the incidences of chronic extrahepatic diseases (eTable 3) may be underestimated due to the semi-competing risks.

### Sensitivity analysis of selection bias by treatment censoring

We characterized the natural history of hepatitis C, particularly to identify specific diseases linking HCV infection with mortality. An ideal design is to follow up a group of HCV carriers without administering any treatment, which is both impractical and unethical. In total, 1.1% of the participants received treatment as interferon, ribavirin, or direct-acting drugs. In this study, we implemented three approaches to tackle the condition of HCV carriers receiving treatment. First, we censored the patients once they had started the treatment. However, this approach may underestimate the risks of diseases and mortality if the treated patients have more severe diseases, whereas it may overestimate the risk if the treated patients represent a group with health-seeking behavior. Second, we followed up as if they were untreated by ignoring the treatment. This approach may underestimate the risk because the treatment should prevent the occurrence of disease or mortality. Third, we used IPW to counteract the imbalance between treated and untreated patients. However, the probability of receiving treatment was modeled only on the basis of baseline characteristics without follow-up information, which is a limitation of this study. We additionally examined the survival probability in treated and untreated patients, and probabilities were similar (eFigure 7). Hence, the second and third approaches (eTable 5, eFigure 5, and eTable 6) revealed similar results with those in the main text, suggesting that the effect of the selection bias, if any, was minimal.

### Strengths and limitations

The strengths of our study include the use of a prospective design with a large sample size and a long-term follow-up (>25 years), the comprehensive determination of disease incidence and mortality through linkage to a nationwide health database, and causal interpretations by performing novel mediation analyses. In addition to the aforementioned potential confounding and selection bias, our study has the following limitations. The covariates were collected only at baseline, which may not be optimal for accounting for time-varying confounding, if any, or adjusting for selection bias by using the time-dependent IPW approach. Moreover, our analyses were only limited to disease prevalence between 10% and 90%. The size of our study sample is still not large enough to study the potential mediation of rare diseases, such as Hodgkin lymphoma and cryoglobulinemia,^29^ or very common diseases. Finally, although the validity of disease determination by using the NHIRD was studied,^30^ potential misclassification may not be excluded. If the misclassification was nondifferential with respect to HCV status, the mediation effect may be underestimated.

### Conclusions

Our study characterized the natural history of HCV infection: 54.1% of HCV-induced mortality was mediated by hepatic diseases and 45.9% by extrahepatic manifestations, including septicemia (25.2%), renal disease (16.7%), blood/immune diseases (12.2%), gallbladder diseases (9.7%), endocrine diseases (9.6%), endocrine diseases (excluding T2DM; 3.4%), genitourinary diseases (1.1%), and genitourinary diseases (excluding renal disease; 0.5%). The mediation mechanisms were supported by the dose-response relationship of HCV viral load.

## SUPPLEMENTARY MATERIAL

eMaterial 1. The ascertainment of potential mediating diseases

eMaterial 2. Detailed statistical methods

eTable 1. Prevalence of potential mediating diseases

eTable 2. The summary table of covariate balance after inverse probability weighting

eTable 3. Hazard ratios of hepatitis C for disease incidence and mortality

eTable 4. Testing results by using the joint significance test for mediation effects and false discovery rate

eTable 5. Hazard ratios of hepatitis C for disease-specific mortality or disease-mediating mortality

eFigure 1. Illustration of the causal structures of unmeasured confounders *U*_1_ and *U*_2_ in the causal diagram.

*γ_Z_*: *the association of HCV and U*_1_

$\gamma_{N_{1}}$ : *the association of mediating disease and U*_1_

*α_U_*: *the association of mortality and U*_1_

$\gamma_{Z}^{\prime }$ : *the association of HCV and U*_2_

*β_U_*: *the association of mediating disease and U*_2_

eFigure 2. Survival ratio in disease-mediating HCV-induced mortality in women stratified by HCV viral load. The survival ratio was estimated using the nonparametric causal mediation estimator with IPW adjustment for the age group (30–39 [reference], 40–49, 50–59, and 60–65 years), sex (reference: women), alcohol consumption (reference: yes), cigarette smoking (reference: yes), ALT (<15 [reference], 15–44, and ≥45 IU/L), interaction between the age group (40–49 years) and sex, interaction between the age group (40–49 years), and ALT levels (15–44 IU/L), interaction between sex and alcohol consumption, interaction between sex and ALT levels (≥45 IU/L), interaction between cigarette smoking and alcohol consumption, and interaction between cigarette smoking and ALT levels (15–44 IU/L). Other disorders of the liver: disorders of the liver excluding fibrosis and cirrhosis of the liver

eFigure 3. Survival ratio in disease–mediating HCV-induced mortality in men stratified by HCV viral load. The survival ratio was estimated using the nonparametric causal mediation estimator with IPW adjustment for the age group (30–39 [reference], 40–49, 50–59, and 60–65 years), sex (reference: women), alcohol consumption (reference: yes), cigarette smoking (reference: yes), ALT (<15 [reference], 15–44, and ≥45 IU/L), interaction between the age group (40–49 years) and sex, interaction between the age group (40–49 years), and ALT levels (15–44 IU/L), interaction between sex and alcohol consumption, interaction between sex and ALT levels (≥45 IU/L), interaction between cigarette smoking and alcohol consumption, and interaction between cigarette smoking and ALT levels (15–44 IU/L). Other disorders of the liver: disorders of the liver excluding fibrosis and cirrhosis of the liver, and fatty liver; renal disease: nephritis, nephrotic syndrome, and nephrosis

eFigure 4. Sensitivity analysis of the mediation effect of HCV-induced mortality through the 12 identified mediating diseases

eFigure 5. HCV-induced mortality by different mediating diseases without censoring for HCV treatment. IPW adjustment was same as that in Figure 3. The proportion of mediation was determined by computing the cumulative hazard difference and calculated as the mediation effect divided by the total effect. Confidence intervals of the mediation proportion were truncated at 0 if they were <0. Other disorders of the liver: excluding fibrosis and cirrhosis of the liver, and fatty liver; renal disease: nephritis, nephrotic syndrome, and nephrosis

eFigure 6. HCV-induced mortality by different mediating diseases with censoring for HCV treatment and inverse probability weighting adjustment. IPW adjustment was the same as that in Figure 3. The proportion of mediation was determined by computing the cumulative hazard difference and calculated as the mediation effect divided by the total effect. Confidence intervals of the mediation proportion were truncated at 0 if they were <0. Other disorders of the liver: excluding fibrosis and cirrhosis of the liver, and fatty liver; renal disease: nephritis, nephrotic syndrome, and nephrosis

eFigure 7. Survival probability of treated and untreated HCV positive participants. The follow-up period for the treated participants started from the day of treatment. In the left panel, the survival probability was estimated using the Cox proportional hazard model with covariate adjustment for the age group (30–39 [reference], 40–49, 50–59, and 60–65 years, at the treatment if treated), sex (reference: women), alcohol consumption (reference: yes), cigarette smoking (reference: yes), ALT (<15 [reference], 15–44, and ≥45 IU/L); in the right panel, it was estimated using the Kaplan-Meier estimator with IPW adjustment for the tendency of HCV treatment.
