# Corrigendum: “Causal Mediation Analyses for the Natural Course of Hepatitis C: A Prospective Cohort Study”

**DOI:** 10.2188/jea.JE20250177

**Published:** 2025-09-05

**Authors:** Yi-Ting Huang, Yao-Chun Hsu, Hwai-I Yang, Mei-Hsuan Lee, Tai-Shuan Lai, Chien-Jen Chen, Yen-Tsung Huang

**Affiliations:** 1 Institute of Statistical Science, Academia Sinica, Taipei, Taiwan; 2 Institute of Epidemiology and Preventive Medicine, National Taiwan University, Taipei, Taiwan; 3 Center for Liver Diseases, E-Da Hospital, Kaohsiung, Taiwan; 4 School of Medicine, I-Shou University, Kaohsiung, Taiwan; 5 Department of Medicine, Fu-Jen Catholic University Hospital, Taipei, Taiwan; 6 Institute of Biomedical Informatics, Yangming Campus, National Yang Ming Chiao Tung University, Taipei, Taiwan; 7 Genomics Research Center, Academia Sinica, Taipei, Taiwan; 8 Institute of Clinical Medicine, Yangming Campus, National Yang Ming Chiao Tung University, Taipei, Taiwan; 9 Graduate Institute of Medicine, Kaohsiung Medical University, Kaohsiung, Taiwan; 10 Biomedical Translation Research Center, Academia Sinica, Taipei, Taiwan; 11 Division of Nephrology, Department of Internal Medicine, National Taiwan University Hospital, Taipei, Taiwan; 12 Department of Mathematics, National Taiwan University, Taipei, Taiwan; 13 Department of Applied Mathematics, National Sun Yat-sen University, Kaohsiung, Taiwan

In the original publication of this article,^1^ due to an oversight on the part of the authors, the name of one of the co-authors was incorrectly spelled as “Tai-Hsuan Lai”. The correct spelling is “Tai-Shuan Lai”. We sincerely apologize for this error.
