# Supplementary material for: Causal Mediation Analyses for the Natural Course of Hepatitis C: A Prospective Cohort Study
Source: J Epidemiol. 2025 Jan 5;35(1):21–9. doi: 10.2188/jea.JE20240034 (PMC11637814; doi:10.2188/jea.JE20240034)
Supplement: Supplementary file 1 [file je-35-021-s001.pdf]

eMaterial 1. The ascertainment of potential mediating diseases

Diseases or manifestations as time-to-event mediators were ascertained using the *International Classification of Diseases, Ninth Revision, Clinical Modification* and *International Classification of Diseases, Tenth Revision, Clinical Modification* codes from the inpatient and outpatient records of the National Health Insurance Research Database (NHIRD). To avoid vague definition in practice, if participants were diagnosed as having a disease as outpatients twice in life or as inpatients once, they would be defined as having this disease at the date of its first diagnosis. For mild and common diseases such as hypertension and type 2 diabetes mellitus (T2DM), related prescribing medications, also linked through the NHIRD, were used as an additional criterion for the outpatient record. Prescribing medications were ascertained by the drug code of the National Health Insurance. For severe diseases including the top 10 causes of mortality in Taiwan such as neoplasms, autoimmune diseases, hemolytic anemia, Crohn's disease, multiple sclerosis, myasthenia gravis, polymyalgia rheumatica, polymyositis/dermatomyositis, primary biliary cirrhosis, rheumatoid arthritis, Sjogren’s syndrome, systemic lupus erythematosus, systemic sclerosis, ulcerative colitis, Wegener’s granulomatosis, ischemic stroke, hemorrhagic stroke, and end-stage renal disease, the incidence was based on a one-time diagnosis (inpatient or outpatient) combined with data linkage to the National Cancer Registry or the Catastrophic Illness Data.

List of disease definition by ICD code

No	Classification	Disease	ICD-9-CM	ICD-10-CM
1	Infection	All	001-139	A00-B99
2	Infection	Septicemia	038, 041.9, 790.7, 785.52	A40-A41, A49.9, B96.89, R65.10, R65.11, R65.20, R65.21, R78.81
3	Infection	Lower respiratory tract infection	480-483, 485-487, 510, 513	A48.1, J09-J16, J18.0-J18.1, J18.8-J18.9, J85-J86
4	Infection	Intra-abdominal infection	540-542, 562.01, 562.03, 562.11, 562.13, 566-567, 569.5, 572.0-572.1, 575.0	K35-K37, K57.00-K57.01, K57.12-K57.13, K57.20-K57.21, K57.32-K57.33, K57.40, K57.52, K57.81, K57.92-K57.93, K61, K63.0, K65, K67, K68.11, K68.19, K68.9, K75.0-K75.1, K81.0
5	Infection	Reproductive and urinary tract infection	590, 599.0, 601, 604, 614-616	N10-N12, N13.6, N15.1, N15.9, N16, N28.84-N28.86, N39.2, N41, N45, N51, N70-N77, N94.810
6	Infection	Skin and soft tissue infection	680-686	B78.1, E83.2, K12.2, L01-L05, L08.0, L08.81-L08.82, L08.89, L08.9, L88, L92.8, L98.0, L98.3
7	Infection	Osteomyelitis	711.0, 730	M00, M46.20-M46.28, M46.30-M46.39, M86, M90.80, M90.811-M90.812, M90.819, M90.821-M90.822, M90.829, M90.831-M90.832, M90.839, M80.841-M80.842, M90.849, M90.851-M90.852, M90.859, M90.861-M80.862, M90.869, M90.871-M90.872, M90.879, M90.88-M90.89
8	Infection	Necrotizing fasciitis	728.86	M72.6
9	Infection	Infectious intestinal diseases	001-009	A00-A09
10	Blood/immune	All	280-289	D50-D89
11	Blood/immune	Addison's disease	255.4	E27.1, E27.2
12	Blood/immune	Ankylosing spondylitis	720	M45, M08.1
13	Blood/immune	Celiac disease	579	K90.0
14	Blood/immune	Chronic rheumatic heart disease	393-398	I05-I09
15	Blood/immune	Graves'/hyperthyroidism	242	E05
16	Blood/immune	Immune thrombocytopenic purpura	287.3	D69.3
17	Blood/immune	Localized scleroderma	701	L94.0
18	Blood/immune	Lupoid hepatitis	571.42	K75.4
19	Blood/immune	Pernicious anemia	281	D51.0
20	Blood/immune	Psoriasis	696	L40
21	Blood/immune	Rheumatic fever	390-392, excluding 392.9	I00-02, excluding I02.9
22	Endocrine	All	240-278	E00-E90
23	Endocrine	All (excluding T2DM)	240-278, excluding 250	E00-E90, excluding E08, E09, E10, E11, E13.00, E13.01, E13.11, E13.2, E13.3, E13.4, E13.5, E13.641, E13.8, E13.9
24	Endocrine	Type II diabetes mellitus	250	E08, E09, E10, E11, E13.00, E13.01, E13.11, E13.2, E13.3, E13.4, E13.5, E13.641, E13.8, E13.9
25	Endocrine	Thyroid disease	240-248	E00, E01, E02, E03, E04, E05, E06, E07,

				E35, E89
26	Endocrine	Metabolic syndrome	277	E71, E76, E79, E80, E81, E84, E85, E88
27	Endocrine	Hyperlipidemia	272	E78.5
28	Endocrine	Cryoglobulinemia	273.2	D89.1
29	Mental and behavioral	All	290-319	F00-F99
30	Nervous system	All	320-389	G00-G99
31	Nervous system	Parkinson's disease	332	G20-G21
32	Circulatory system	All	390-459	I00-I99
33	Circulatory system	Rheumatic heart disease	chronic: 393-398; acute: 391.9	chronic: I05-I09; acute: I01.9
34	Circulatory system	Ischemic heart disease (Coronary heart disease)	410-414	I20-I25
35	Circulatory system	Cerebrovascular disease	430-438	I60-I69
36	Circulatory system	Hypertensive heart disease	402	I11
37	Circulatory system	Cardiomyopathy and myocarditis	414.8, 425	I25.5, I42, I43
38	Circulatory system	Atrial fibrillation and flutter	427.3	I48
39	Circulatory system	Aortic aneurysm	441	I71
40	Circulatory system	Peripheral vascular disease	443	I73
41	Circulatory system	Endocarditis	424.90, 424.99, 115.04, 115.14, 115.94, 421.1, 424.91	I38-I39
42	Circulatory system	Heart disease	391-398, 402, 404, 410-416, 420-429	I51.9
43	Circulatory system	Congestive heart failure	428	I50
44	Circulatory system	Hypertensive disease	401-405	I10-I15
45	Circulatory system	Essential (primary) hypertension	401	I10
46	Respiratory system	All	460-519	J00-J99
47	Digestive system	All	520-579	K00-K-93
48	Digestive system	Diseases of esophagus, stomach, and duodenum	530-539	K20-K31
49	Digestive system	Gastroesophageal reflux	530.81	K21.9
50	Digestive system	Appendicitis	540-543	K35-K38
51	Digestive system	Hernia of abdominal cavity	550-553	K40-K46
52	Digestive system	Noninfectious enteritis and colitis	555-558	K50-K52
53	Digestive system	Other diseases of intestines and peritoneum	560-569	K55-K64
54	Digestive system	Other disorders of liver (excluding Fibrosis and cirrhosis of liver)	570-573, excluding 571.5, 571.6	K70-K77, excluding K74
55	Digestive system	Fibrosis and cirrhosis of liver	571.5, 571.6	K74
56	Digestive system	Diseases of pancreas	577	K85-K86
57	Digestive system	Diseases of gallbladder	574-576	K80-K83
58	Digestive system	Fatty liver	571.0, 571.8	K70.0, K76.0, K76.89

59	Digestive system	Non-alcohol fatty liver	571.8	K76.0, K76.89
60	Skin	All	680-709	L00-L99
61	Musculoskeletal	All	710-739	M00-M99
62	Genitourinary	All	580-629	N00-N99
63	Genitourinary	All (excluding renal diseases)	580-629, excluding 580-589	N00-N99, excluding N00-N29
64	Genitourinary	Renal disease (nephritis, nephrotic syndrome, and nephrosis)	580-589	N00-N29
65	Pregnancy/perinatal/congenital	All	630-679, 740-759, 760–779	O00-Q99
66	Other/External	All	E800-E999	S00-T98, V00-Y98
67	Blood/immune	Autoimmune hemolytic anemia	283	D59.0
68	Blood/immune	Crohn's disease	555	K50
69	Blood/immune	Multiple sclerosis	340	G35
70	Blood/immune	Myasthenia gravis	358	G70.0
71	Blood/immune	Polymyalgia rheumatica	725	M315, M35.3
72	Blood/immune	Polymyositis/dermatomyositis	710.3, 710.4	M33
73	Blood/immune	Primary biliary cirrhosis	571.6	K74.3
74	Blood/immune	Rheumatoid arthritis	714, excluding 714.1, 714.9	M05, M06, M08.0, M08.2
75	Blood/immune	Sjo€gren's syndrome	710.2	M35.0
76	Blood/immune	Systemic lupus erythematosus	710	M32
77	Blood/immune	Systemic sclerosis	710.1, 517.2	M34
78	Blood/immune	Ulcerative colitis	556	K51
79	Blood/immune	Wegener's granulomatosis	446.4	M31.3
80	Circulatory system	Ischemic stroke	436	I67.89
81	Circulatory system	Hemorrhagic stroke	431	I61.9
82	Genitourinary	End-stage renal disease	403.01, 403.11, 403.91, 404.02, 404.03, 404.12, 404.13, 404.92, 404.93, 585	N18.40, N18.5, N18.6, N18.9, I12.0, I12.9, I13.11, I13.2, V42.0, Z94.0
83	Neoplasms	All	140-239	C00-D48
84	Neoplasms	All (excluding liver cancer)	140-239, excluding 155	C00-D48, excluding C22
85	Neoplasms	Trachea, bronchial and lung cancers	162	C33-C34
86	Neoplasms	Liver cancer and cholangiocarcinoma	155	C22
87	Neoplasms	Colon, rectal and anal cancers	153-154	C18-C21
88	Neoplasms	Breast cancer in women	174	C50
89	Neoplasms	Oral cancer	140-141, 143-146, 148-149	C00-C06, C09-C10, C12-C14
90	Neoplasms	Prostate cancer	185	C61
91	Neoplasms	Gastric cancer	151	C16
92	Neoplasms	Pancreatic cancer	157	C25
93	Neoplasms	Esophageal cancer	150	C15

94	Neoplasms	Cervical and unspecified uterine cancers	179-180	C53, C55
95	Neoplasms	Malignant neoplasm of thyroid gland	193	C73
96	Neoplasms	Hodgkin lymphoma	201	C81
97	Neoplasms	Leukemia	204-208	C91-C95

eMaterial 2. Detailed statistical methods

Nonparametric mediation analysis with a time-to-event mediator

First, we denote T_{2i}, T_{1i}, C_i and Z_i as time to death, time to disease, censoring time, and hepatitis C status, respectively, for patient i, $i = 1, \dots, n$ where n is the sample size. We further denote $U_i = \min(T_{2i}, C_i)$, $\delta_i = I(T_{2i} \le C_i)$, $N_{1i}(t) = I(T_{1i} \le t, T_{1i} \le U_i)$, and $N_{2i}(t) = I(U_i \le t, \delta_i = 1)$. z_a and z_b represent different counterfactual interventions. The nonparametric analysis estimated hazards[1] in the population with and without mediating diseases and hepatitis C virus (HCV) at fixed time points (i.e., for $n_1 = 0$ and 1, $\frac{I(\bar{Y}_{n_1}(s|z_a)>0)}{\bar{Y}_{n_1}(s|z_a)} d\bar{N}_{2n_1}(s|z_a)$) and then weighted the HCV/disease–dependent hazard by the HCV-specific disease prevalence among survivors (i.e., $\widehat{w}_{n_1}(t|z_b)$). By summing up the weighted hazard across all time points, we obtained the cumulative hazard:

Cumulative hazard:

$$\begin{aligned}\widehat{\Lambda}_{n_1}(t; z_a, z_b) &= \widehat{\Lambda}_0(t; z_a, z_b) + \widehat{\Lambda}_1(t; z_a, z_b)\\ &= \sum_{n1=0}^{1} \int_0^t \widehat{w}_{n_1}(s|z_b) \frac{I\left(\bar{Y}_{n_1}(s|z_a) > 0\right)}{\bar{Y}_{n_1}(s|z_a)} d\bar{N}_{2n_1}(s|z_a),\end{aligned}$$

where $\widehat{w}_{n_1}(t|z_b) = \dfrac{\sum_{i=1}^{m} I(N_{1i}(t^-) = n_1, U_i \ge t, Z_i = z_b)}{\sum_{i=1}^{m} I(U_i \ge t, Z_i = z_b)}$,

$\bar{Y}_{n_1}(t|Z) = \sum_{i=1}^{m} Y_{n_1 i}(t|Z)$,

$\bar{N}_{2n_1}(t|Z) = \sum_{i=1}^{m} N_{2n_1 i}(t|Z)$,

$Y_{n_1 i}(t|Z) = I(U_i \ge t, Z_i = Z, N_{1i}(t^-) = n_1)$ is the at-risk process of subject i at time t with the mediating disease status being $n_1 \in \{0,1\}$,

$N_{2n_1 i}(t|Z) = \mathrm{I}(U_i \le t, \delta_i = 1, Z_i = Z, N_{1i}(t^-) = n_1)$ is the (mortality) event process of subject i at time t with the mediating disease status being $n_1 \in \{0,1\}$

By plugging-in the different combinations of HCV status into the cumulative hazard and considering differences (or ratios), we estimated the mediation effect and alternative effect in the scale of the cumulative hazard difference (or cumulative hazard ratio):

Mediation effect:

$$\text{Risk difference}_{\text{mediation}} \coloneqq \widehat{\Lambda}(t; z_a = 1, z_b = 1) - \widehat{\Lambda}(t; z_a = 1, z_b = 0)$$

$$\text{Risk ratio}_{\text{mediation}} \coloneqq \frac{\widehat{\Lambda}(t; z_a = 1, z_b = 1)}{\widehat{\Lambda}(t; z_a = 1, z_b = 0)}$$

$$\text{Survival ratio}_{\text{mediation}} \coloneqq exp\left(-\widehat{\Lambda}(t; z_a = 1, z_b = 1) + \widehat{\Lambda}(t; z_a = 1, z_b = 0)\right)$$

Alternative effect:

$$\text{Risk difference}_{\text{alternative}} \coloneqq \widehat{\Lambda}(t; z_a = 1, z_b = 0) - \widehat{\Lambda}(t; z_a = 0, z_b = 0)$$

$$\text{Risk ratio}_{\text{alternative}} \coloneqq \frac{\widehat{\Lambda}(t; z_a = 1, z_b = 0)}{\widehat{\Lambda}(t; z_a = 0, z_b = 0)}$$

$$\text{Survival ratio}_{\text{alternative}} \coloneqq exp\left(-\widehat{\Lambda}(t; z_a = 1, z_b = 0) + \widehat{\Lambda}(t; z_a = 0, z_b = 0)\right)$$

The proportion of mediation can be calculated as follows.

Proportion of mediation:

$$\frac{\text{mediation effect}}{\text{mediation effect+alternative effect}} = \frac{\int_0^\infty \left\{exp\left(-\widehat{\Lambda}(t; z_a = 1, z_b = 1) + \widehat{\Lambda}(t; z_a = 1, z_b = 0)\right)\right\} dt}{\int_0^\infty \left\{exp\left(-\widehat{\Lambda}(t; z_a = 1, z_b = 1) + \widehat{\Lambda}(t; z_a = 0, z_b = 0)\right)\right\} dt}$$

Sensitivity analysis

Two sets of sensitivity analyses were performed to examine the robustness of the results of the mediation analysis. In the first part, we examined the influence of unmeasured confounders by using numerical simulation. We artificially generated two confounders illustrated by causal diagrams in Figure S1. One confounder was associated with the mediator and outcome, and the other was associated with the exposure, mediator, and outcome (eFigure 1). We investigated five scenarios by setting different combinations of confounding effects (indicated by γ in the following, ranging from −1 to +1) of the two confounders.

Scenario 1: $\alpha_U = \beta_U = \gamma, \gamma_Z = \gamma_{N_1} = \gamma'_Z = |\gamma|\ where\ -1 < \gamma < 1$

Scenario 2: $\alpha_U = \beta_U = log(1.5), \gamma_Z = \gamma_{N_1} = \gamma'_Z = |\gamma|\ where\ 0 < \gamma < 1$

Scenario 3: $\alpha_U = \beta_U = -log(1.5), \gamma_Z = \gamma_{N_1} = \gamma'_Z = |\gamma|\ where\ 0 < \gamma < 1$

Scenario 4: $\alpha_U = \beta_U = \gamma, \gamma_Z = \gamma_{N_1} = \gamma'_Z = 0.2\ where\ 0 < \gamma < 1$

Scenario 5: $\alpha_U = \beta_U = 0, \gamma_Z = \gamma_{N_1} = \gamma'_Z = \gamma\ where\ -1 < \gamma < 1$

Sensitivity analysis indicated how the magnitude of mediation effects can be altered by two simulated unmeasured confounders in Figure S4 by changing the confounding effect from low (lines in yellow colors) to high (lines in pink colors). For instance, air pollution, with a survival ratio of approximately 1.5 in Scenario 2, serves as an unmeasured confounder[2,3]. Across the wide range of confounding, the corrected effects of all mediating diseases were not changed qualitatively; that is, having effects in the same direction as the original effects (line in red color).

In the second part, we evaluated the potential selection bias induced by censoring participants who received treatment against HCV. Furthermore, we conducted two additional analyses. In the first analysis, we did not censor the participants who received any therapeutics for HCV infection. In the second analysis, we censored the participants who received HCV treatment and performed an additional inverse probability weighting (IPW) adjustment for the propensity of receiving treatment by covariates measured at the baseline. In particular, the propensity to receive treatment for HCV was modeled by performing stepwise logistic regression to select age groups, sex, alanine aminotransferase (ALT) levels, and their potential cross-product interactions.

REFERENCES

1. Huang YT. Causal mediation of semicompeting risks. Biometrics. 2021;77(4):1143-1154.

2. Ancona C, Badaloni C, Mataloni F, et al. Mortality and morbidity in a population exposed to multiple sources of air pollution: A retrospective cohort study using air dispersion models. Environmental research. 2015;137:467-474.

3. Turner MC, Krewski D, Diver WR, et al. Ambient air pollution and cancer mortality in the cancer prevention study II. Environmental health perspectives. 2017;125(8):087013.

eTable 1. Prevalence of potential mediating diseases

	HCV (–) & HBV (–) (N=18,038)		HCV (+) (N=934)		Total (N=18,972)	
Potential mediating disease	# of disease	Prevalence	# of Disease	Prevalence	# of disease	Prevalence
All infection diseases	17,589	0.975	919	0.984	18,508	0.976
Septicemia	2,749	0.152	219	0.234	2,968	0.156
Lower respiratory tract infection	8,296	0.46	445	0.476	8,741	0.461
Intra-abdominal infection	1,352	0.075	104	0.111	1,456	0.077
Reproductive and urinary tract infection	9,167	0.508	506	0.542	9,673	0.51
Skin and soft tissue infection	8,205	0.455	421	0.451	8,626	0.455
Osteomyelitis	568	0.031	41	0.044	609	0.032
Necrotizing fasciitis	102	0.006	8	0.009	110	0.006
Infectious intestinal diseases	6,083	0.337	393	0.421	6,476	0.341
All blood/immune diseases	4,601	0.255	326	0.349	4,927	0.26
Addison's disease	19	0.001	0	0	19	0.001
Ankylosing spondylitis	189	0.01	7	0.007	196	0.01
Celiac disease	1	0	0	0	1	0
Chronic rheumatic heart disease	615	0.034	46	0.049	661	0.035
Graves'/hyperthyroidism	780	0.043	35	0.037	815	0.043

Immune thrombocytopenic purpura	33	0.002	1	0.001	34	0.002
Localized scleroderma	9	0	0	0	9	0
Lupoid hepatitis	4,506	0.25	596	0.638	5,102	0.269
Pernicious anemia	15	0.001	1	0.001	16	0.001
Psoriasis	334	0.019	9	0.01	343	0.018
Rheumatic fever	5	0	1	0.001	6	0
All endocrine diseases	14,457	0.801	801	0.858	15,258	0.804
All endocrine diseases (excluding T2DM)	12,638	0.701	682	0.73	13,320	0.702
Type II diabetes mellitus	4,830	0.268	310	0.332	5,140	0.271
Thyroid disease	2,003	0.111	114	0.122	2,117	0.112
Metabolic syndrome	7,433	0.412	485	0.519	7,918	0.417
Hyperlipidemia	1,815	0.101	67	0.072	1,882	0.099
Cryoglobulinemia	1	0	0	0	1	0
All mental and behavioral diseases	10,818	0.6	589	0.631	11,407	0.601
All nervous system diseases	16,690	0.925	873	0.935	17,563	0.926
Parkinson’s disease	967	0.054	56	0.06	1,023	0.054
All circulatory system diseases	15,192	0.842	817	0.875	16,009	0.844
Rheumatic heart disease	618	0.034	46	0.049	664	0.035
Ischemic heart disease	6,885	0.382	372	0.398	7,257	0.383

Cerebrovascular disease	5,268	0.292	276	0.296	5,544	0.292
Hypertensive heart disease	1,994	0.111	71	0.076	2,065	0.109
Cardiomyopathy and myocarditis	591	0.033	37	0.04	628	0.033
Atrial fibrillation and flutter	1,175	0.065	76	0.081	1,251	0.066
Aortic aneurysm	132	0.007	6	0.006	138	0.007
Peripheral vascular disease	1,190	0.066	68	0.073	1,258	0.066
Endocarditis	269	0.015	18	0.019	287	0.015
Heart disease	10,373	0.575	581	0.622	10,954	0.577
Congestive heart failure	2,618	0.145	171	0.183	2,789	0.147
Hypertensive disease	7,774	0.431	290	0.31	8,064	0.425
Essential (primary) hypertension	6,256	0.347	367	0.393	6,623	0.349
All respiratory system diseases	17,613	0.976	911	0.975	18,524	0.976
All digestive system diseases	17,114	0.949	908	0.972	18,022	0.95
Diseases of esophagus, stomach, and duodenum	15,309	0.849	844	0.904	16,153	0.851
Gastroesophageal reflux	1,494	0.083	82	0.088	1,576	0.083
Appendicitis	422	0.023	24	0.026	446	0.024
Hernia of abdominal cavity	1,616	0.09	88	0.094	1,704	0.09
Noninfectious enteritis and colitis	8,889	0.493	500	0.535	9,389	0.495
Other diseases of intestines and peritoneum	11,525	0.639	633	0.678	12,158	0.641

Fibrosis and cirrhosis of liver	430	0.024	201	0.215	631	0.033
Other disorders of liver	6,346	0.352	672	0.719	7,018	0.37
Diseases of pancreas	460	0.026	43	0.046	503	0.027
Diseases of gallbladder	2,559	0.142	214	0.229	2,773	0.146
Fatty liver disease	993	0.055	91	0.097	1,084	0.057
Non-alcoholic fatty liver disease	899	0.05	86	0.092	985	0.052
All skin diseases	16,192	0.898	824	0.882	17,016	0.897
All musculoskeletal diseases	17,089	0.947	868	0.929	17,957	0.947
All genitourinary diseases	14,922	0.827	784	0.839	15,706	0.828
All genitourinary diseases (excluding renal diseases)	13,975	0.775	733	0.785	14,708	0.775
Renal disease	7,451	0.413	398	0.426	7,849	0.414
All pregnancy/perinatal/congenital diseases	1,762	0.098	103	0.11	1,865	0.098
All other/external diseases	16,709	0.926	852	0.912	17,561	0.926
Autoimmune hemolytic anemia	0	0.000	0	0.000	0	0.000
Crohn's disease	≤ 3	-	0	0.000	-	-
Multiple sclerosis	≤ 3	-	0	0.000	-	-
Myasthenia gravis	17	0.001	1	0.001	18	0.001
Polymyalgia rheumatica	0	0.000	0	0.000	0	0.000
Polymyositis/dermatomyositis	≤ 3	-	1	0.001	-	-

Primary biliary cirrhosis	≤ 3	-	≤ 3	-	-	-
Rheumatoid arthritis	104	0.006	7	0.007	111	0.006
Sjogren's syndrome	54	0.003	≤ 3	-	-	-
Systemic lupus erythematosus	23	0.001	1	0.001	24	0.001
Systemic sclerosis	7	0.000	1	0.001	8	0.000
Ulcerative colitis	7	0.000	1	0.001	8	0.000
Wegener's granulomatosis	0	0.000	0	0.000	0	0.000
Ischemic stroke	0	0.000	0	0.000	0	0.000
Hemorrhagic stroke	0	0.000	0	0.000	0	0.000
End-stage renal disease	361	0.02	33	0.035	394	0.021
All neoplasms	2,704	0.15	253	0.271	2,957	0.156
All neoplasms (excluding liver cancer)	2,571	0.143	143	0.153	2,714	0.143
Trachea, bronchial and lung cancers	397	0.022	19	0.02	416	0.022
Liver cancer and cholangiocarcinoma	153	0.008	127	0.136	280	0.015
Colon, rectal and anal cancers	448	0.025	33	0.035	481	0.025
Breast cancer in women	316	0.034	11	0.021	327	0.033
Oral cancer	106	0.006	5	0.005	111	0.006
Prostate cancer	217	0.025	12	0.03	229	0.025
Gastric cancer	134	0.007	12	0.013	146	0.008

Pancreatic cancer	68	0.004	7	0.007	75	0.004
Esophageal cancer	40	0.002	2	0.002	42	0.002
Cervical and unspecified uterine cancers	219	0.024	12	0.022	231	0.023
Cancer of thyroid gland	44	0.002	3	0.003	47	0.002
Hodgkin lymphoma	0	0	0	0	0	0
Leukemia	0	0	0	0	0	0

HBV, hepatitis B virus; HCV, hepatitis C virus; T2DM, type 2 diabetes mellitus.

Data are presented before censoring when prescribing HCV drugs.

Due to the policy of personal data protection by the NHIRD, we displayed "≤ 3" if the number of having the potential mediating disease was lower than or equal to 3 persons. The prevalence was labeled as "-" to represent that the disease had less than or equal to 3 persons in the HCV (–) group, HCV (+) group, or the difference between HCV (–) and HCV (+). Some of cells were shown "-" because the exact value could not be calculated.

Other disorders of the liver: disorders of liver excluding fibrosis and cirrhosis of the liver, and fatty liver; renal disease: nephritis, nephrotic syndrome, and nephrosis; inf: infinity

eTable 2. The summary table of covariate balance after inverse probability weighting

	HCV (−) and HBV (−) (N=17,962.64)	HCV (+) (N=538.49)	*P* value
Age, years			0.698
30–39	5,120.50 (28.51)	162.05 (30.09)	
40–49	4,728.98 (26.33)	136.65 (25.38)	
50–59	5,508.01 (30.66)	160.06 (29.72)	
60–65	2,605.15 (14.50)	79.73 (14.81)	
Sex			0.046
Women	9,327.10 (51.92)	256.34 (47.60)	
Men	8,635.54 (48.08)	282.15 (52.40)	
Cigarette smoking			0.227
Yes	4,967.42 (27.65)	162.27 (30.13)	
No	12,995.22 (72.35)	376.22 (69.87)	
Drinking consumption			0.200
Yes	1,814.71 (10.10)	65.75 (12.21)	
No	16,147.93 (89.90)	472.75 (87.79)	
ALT, IU/L			0.795

<15	12,967.48 (72.19)	393.99 (73.16)	
15–44	4,505.50 (25.08)	130.83 (24.30)	
≥45	489.66 (2.73)	13.68 (2.54)	
HCV RNA viral load, IU/mL			0.269
Undetectable	-	216.35 (40.18)	
Detectable	-	322.14 (59.82)	

ALT, alanine transaminase; HBV, hepatitis B virus; HCV, hepatitis C virus.

eTable 3. Hazard ratios of hepatitis C for disease incidence and mortality

Mediating disease	Crude incidence rate ratio (95% CI)	HCV (+) HR (95% CI)	HCV RNA viral load, HR (95% CI)	
			Undetectable	Detectable
Intrahepatic manifestation	9.94 (9.79–10.10)	7.01 (5.70–8.62)	2.27 (1.35–3.84)	11.24 (9.03–13.99)
Septicemia	1.75 (1.53–2.01)	1.55 (1.28–1.89)	1.20 (0.83–1.74)	1.88 (1.51–2.33)
Lower respiratory tract infection	1.18 (1.07–1.30)	1.11 (0.97–1.26)	1.00 (0.81–1.24)	1.19 (1.02–1.40)
Intra-abdominal infection	1.68 (1.37–2.05)	1.56 (1.21–2.01)	0.81 (0.49–1.32)	2.22 (1.65–2.97)
Reproductive and urinary tract infection	1.20 (1.10–1.32)	1.03 (0.92–1.16)	1.19 (0.99–1.42)	0.92 (0.80–1.07)
Skin and soft tissue infection	1.14 (1.03–1.26)	1.06 (0.93–1.21)	1.06 (0.86–1.30)	1.06 (0.90–1.26)
Infectious intestinal diseases	1.53 (1.38–1.69)	1.32 (1.15–1.52)	1.38 (1.11–1.71)	1.27 (1.06–1.52)
All blood/immune diseases	1.59 (1.42–1.78)	1.36 (1.17–1.58)	1.06 (0.82–1.36)	1.63 (1.36–1.95)
Lupoid hepatitis	3.86 (3.54–4.20)	3.30 (2.91–3.73)	1.88 (1.51–2.34)	4.84 (4.21–5.57)
All endocrine diseases	1.34 (1.25–1.44)	1.28 (1.15–1.42)	1.25 (1.07–1.47)	1.30 (1.13–1.49)
All endocrine diseases (excluding T2DM)	1.25 (1.16–1.35)	1.24 (1.11–1.37)	1.28 (1.09–1.50)	1.21 (1.05–1.39)
Type II diabetes mellitus	1.45 (1.29–1.63)	1.05 (0.90–1.23)	0.98 (0.76–1.26)	1.11 (0.91–1.35)
Thyroid disease	1.24 (1.03–1.50)	1.11 (0.87–1.42)	1.29 (0.91–1.82)	0.97 (0.69–1.36)
Metabolic syndrome	1.57 (1.43–1.72)	1.17 (1.03–1.34)	1.09 (0.87–1.36)	1.24 (1.05–1.46)
All mental and behavioral diseases	1.22 (1.12–1.32)	1.04 (0.93–1.17)	0.95 (0.78–1.16)	1.12 (0.97–1.28)
All circulatory system diseases	1.21 (1.12–1.29)	1.00 (0.90–1.10)	0.96 (0.82–1.11)	1.03 (0.90–1.18)
Ischemic heart disease	1.20 (1.08–1.33)	0.96 (0.84–1.11)	0.90 (0.72–1.13)	1.01 (0.84–1.22)
Cerebrovascular disease	1.15 (1.02–1.30)	0.94 (0.79–1.10)	0.91 (0.70–1.20)	0.95 (0.78–1.17)

Heart disease	1.27 (1.17–1.38)	1.00 (0.89–1.12)	0.91 (0.75–1.09)	1.07 (0.93–1.24)
Congestive heart failure	1.45 (1.24–1.69)	1.13 (0.92–1.39)	0.95 (0.69–1.29)	1.29 (0.99–1.69)
Hypertensive disease	0.81 (0.72–0.91)	0.88 (0.75–1.02)	0.91 (0.73–1.13)	0.84 (0.67–1.05)
Essential (primary) hypertension	1.31 (1.18–1.45)	1.12 (0.96–1.30)	1.07 (0.84–1.36)	1.16 (0.96–1.40)
Noninfectious enteritis and colitis	1.26 (1.15–1.38)	1.19 (1.05–1.34)	1.21 (1.01–1.46)	1.17 (1.00–1.37)
Other diseases of intestines and peritoneum	1.22 (1.13–1.33)	1.08 (0.97–1.21)	1.05 (0.89–1.24)	1.11 (0.97–1.28)
Fibrosis and cirrhosis of liver	10.70 (9.05–12.65)	7.73 (6.22–9.60)	2.37 (1.34–4.19)	12.44 (9.91–15.61)
Other disorders of liver	3.15 (2.91–3.41)	2.83 (2.51–3.19)	1.80 (1.47–2.20)	3.96 (3.45–4.54)
Diseases of gallbladder	1.88 (1.64–2.16)	1.50 (1.25–1.81)	1.08 (0.76–1.52)	1.86 (1.50–2.31)
All skin diseases	1.06 (0.99–1.14)	1.04 (0.94–1.14)	0.99 (0.85–1.15)	1.07 (0.96–1.20)
All genitourinary diseases	1.15 (1.07–1.24)	1.08 (0.98–1.18)	1.10 (0.95–1.28)	1.06 (0.94–1.19)
All genitourinary diseases (excluding renal diseases)	1.13 (1.05–1.22)	1.04 (0.94–1.14)	1.08 (0.94–1.24)	1.00 (0.89–1.14)
Renal disease	1.21 (1.10–1.34)	1.28 (1.11–1.47)	1.10 (0.88–1.37)	1.47 (1.22–1.76)
All pregnancy/perinatal/congenital diseases	1.28 (1.05–1.56)	1.23 (0.94–1.60)	1.01 (0.65–1.56)	1.42 (1.03–1.97)
All neoplasms	2.08 (1.83–2.36)	1.67 (1.40–1.99)	0.98 (0.68–1.41)	2.30 (1.89–2.80)
All neoplasms (excluding liver cancer)	1.21 (1.03–1.44)	1.16 (0.93–1.45)	0.91 (0.61–1.36)	1.38 (1.06–1.80)
Liver cancer and cholangiocarcinoma	18.40 (14.54–23.28)	10.87 (8.16–14.48)	1.99 (0.88–4.47)	18.94 (14.04–25.54)
All-cause mortality	2.05 (1.94–2.15)	1.74 (1.51–2.02)	1.22 (0.93–1.61)	2.22 (1.87–2.63)
All-cause mortality (excluding other disorders of liver)	1.64 (1.53–1.76)	1.37 (1.19–1.57)	1.03 (0.83–1.28)	1.69 (1.43–1.99)
All-cause mortality (excluding fibrosis and cirrhosis of liver)	1.93 (1.83–2.03)	1.54 (1.35–1.76)	1.05 (0.85–1.30)	2.00 (1.72–2.32)

All-cause mortality (excluding liver cancer and cholangiocarcinoma)	1.48 (1.36–1.60)	1.28 (1.11–1.48)	1.04 (0.83–1.29)	1.50 (1.26–1.79)
All-cause mortality (excluding intrahepatic manifestation)	1.93 (1.83–2.03)	1.54 (1.35–1.76)	1.05 (0.85–1.30)	2.00 (1.72–2.32)
All-cause mortality (excluding liver related)	1.74 (1.63–1.85)	1.45 (1.27–1.67)	1.05 (0.84–1.30)	1.83 (1.55–2.15)

CI, confidence interval; HCV, hepatitis C virus; HR, hazard ratio; T2DM, type 2 diabetes mellitus.

The reference group of HCV positive is HCV and hepatitis B virus (HBV) negative.

Adjusted hazard ratios were estimated using the Cox proportional hazard model with IPW adjustment for the age group (30–39 [reference], 40–49, 50–59, and 60–65 years), sex (reference: women), alcohol consumption (reference: yes), cigarette smoking (reference: yes), ALT levels (<15 [reference], 15–44, and ≥45 IU/L), interaction between the age group (40–49 years) and sex, interaction between the age group (40–49 years) and ALT levels (15–44 IU/L), interaction between sex and alcohol consumption, interaction between sex and ALT levels (≥45 IU/L), interaction between cigarette smoking and alcohol consumption, and interaction between cigarette smoking and ALT levels (15–44 IU/L).

Other disorders of the liver: disorders of the liver excluding fibrosis and cirrhosis of the liver, and fatty liver; renal disease: nephritis, nephrotic syndrome, and nephrosis

eTable 4. Testing results by using the joint significance test for mediation effects and false discovery rate

Potential mediating diseases	*p* value			*q* value		
	All	Women	Men	All	Women	Men
Septicemia	0.072791	0.107214	0.199159	0.097032	0.123863	0.168355
Lower respiratory tract infection	0.157669	0.307658	0.354985	0.151794	0.201528	0.201528
Intra-abdominal infection	0.418783	0.134748	0.770802	0.213447	0.137358	0.296832
Reproductive and urinary tract infection	0.695108	0.846812	0.464230	0.280414	0.312226	0.226613
Skin and soft tissue infection	0.387727	0.286716	0.695805	0.201528	0.201528	0.280414
Infectious intestinal diseases	0.725794	0.314361	0.969281	0.285852	0.201528	0.330465
All blood/immune diseases	0.034286	0.063937	0.232999	0.079220	0.088638	0.187800
Lupoid hepatitis	0.389541	0.597208	0.593307	0.201528	0.258729	0.258729
All endocrine diseases	0.001170	0.127863	0.007346	0.009398	0.134289	0.031823
All endocrine diseases (excluding T2DM)	0.053837	0.595469	0.039462	0.088638	0.258729	0.080454
Type II diabetes mellitus	0.383814	0.079514	0.541880	0.201528	0.098423	0.253088
Thyroid disease	0.374470	0.972558	0.279378	0.201528	0.330465	0.201528
Metabolic syndrome	0.127611	0.015667	0.943786	0.134289	0.054298	0.330406
All mental and behavioral diseases	0.906824	0.654854	0.876503	0.322882	0.273449	0.318355
All circulatory system diseases	0.627484	0.389583	0.171663	0.268490	0.201528	0.156938
Ischemic heart disease	0.716475	0.175965	0.367384	0.285425	0.156938	0.201528
Cerebrovascular disease	0.198712	0.346345	0.544212	0.168355	0.201528	0.253088
Heart disease	0.881806	0.339018	0.752914	0.318355	0.201528	0.293201
Congestive heart failure	0.323211	0.639597	0.328140	0.201528	0.270335	0.201528
Hypertensive disease	0.810054	0.307918	0.116358	0.305166	0.201528	0.130090
Essential (primary) hypertension	0.349765	0.061503	0.912978	0.201528	0.088638	0.322882
Noninfectious enteritis and colitis	0.343523	0.307325	0.461918	0.201528	0.201528	0.226613
Other diseases of intestines and peritoneum	0.293322	0.959696	0.154246	0.201528	0.330465	0.151794
Fibrosis and cirrhosis of liver	0.000006	0.000960	0.001356	0.000207	0.009398	0.009398
Other disorders of liver	0.057620	0.018927	0.481715	0.088638	0.058029	0.231883
Diseases of gallbladder	0.075749	0.547676	0.061021	0.097235	0.253088	0.088638
All skin diseases	0.672935	0.206036	0.807409	0.277654	0.170021	0.305166
All genitourinary diseases	0.030670	0.846371	0.083626	0.075926	0.312226	0.099943
All genitourinary diseases (excluding renal diseases)	0.038257	0.265498	0.060658	0.080454	0.201528	0.088638
Renal disease	0.001657	0.022545	0.020092	0.009572	0.060105	0.058029
All pregnancy/perinatal/congenital	0.568144	0.555013	0.365586	0.255727	0.253104	0.201528

diseases						
All neoplasms	0.046879	0.176596	0.055599	0.088638	0.156938	0.088638
All neoplasms (excluding liver cancer)	0.443872	0.349007	0.385436	0.222956	0.201528	0.201528
Liver cancer and cholangiocarcinoma	0.000295	0.001953	0.010790	0.005112	0.009671	0.041551

T2DM, type 2 diabetes mellitus.

The q values were calculated using the method by Storey et al..

Other disorders of the liver: disorders of the liver excluding fibrosis and cirrhosis of the liver, and fatty liver; renal disease: nephritis, nephrotic syndrome, and nephrosis

eTable 5. Hazard ratios of hepatitis C for disease-specific mortality or disease-mediating mortality

Significant mediating disease	Sensitivity analysis[a]		Sensitivity analysis[b]	
	Disease-specific mortality<br>CHR (95% CI)	Disease-mediating mortality<br>25-years CHR (95% CI)	Disease-specific mortality<br>CHR (95% CI)	Disease-mediating mortality<br>25-years CHR (95% CI)
All				
All-cause mortality	1.64 (1.42–1.90)	1.69 (1.43–1.92)	1.84 (1.58–2.15)	1.89 (1.59–2.16)
Septicemia	0.70 (0.23–2.16)	1.13 (1.03–1.24)	0.74 (0.24–2.28)	1.15 (1.04–1.27)
All blood/immune diseases	-	1.09 (1.03–1.15)	-	1.07 (1.01–1.14)
All endocrine diseases	1.18 (0.71–1.95)	1.04 (1.02–1.07)	1.23 (0.74–2.05)	1.04 (1.01–1.06)
All endocrine diseases (excluding T2DM)	0.67 (0.31–1.41)	1.02 (1.00–1.05)	0.70 (0.32–1.51)	1.02 (1.00–1.04)
Fibrosis and cirrhosis of liver	12.53 (5.56–28.23)	1.32 (1.23–1.42)	14.61 (6.43–33.23)	1.31 (1.22–1.41)
Other disorders of liver	3.71 (1.58–8.67)	1.08 (0.97–1.19)	4.31 (1.79–10.40)	1.13 (1.04–1.23)
Diseases of gallbladder	-	1.05 (1.02–1.09)	-	1.04 (1.00–1.08)
All genitourinary diseases	1.83 (1.06–3.14)	1.01 (1.00–1.02)	2.09 (1.21–3.59)	1.01 (0.99–1.02)
All genitourinary diseases (excluding renal diseases)	2.02 (1.08–3.81)	1.01 (0.99–1.02)	2.32 (1.23–4.39)	1.01 (0.99–1.02)
Renal disease	1.30 (0.51–3.29)	1.08 (1.05–1.13)	1.47 (0.60–3.61)	1.08 (1.04–1.12)
All neoplasms	2.35 (1.90–2.90)	1.12 (1.03–1.21)	2.63 (2.11–3.28)	1.13 (1.03–1.23)
Liver cancer and cholangiocarcinoma	10.92 (7.89–15.12)	1.22 (1.16–1.30)	12.12 (8.65–17.00)	1.21 (1.15–1.28)
Women				
All-cause mortality	1.67 (1.37–2.05)	1.74 (1.41–2.09)	1.83 (1.49–2.26)	1.87 (1.50–2.28)
All blood/immune diseases	-	1.09 (1.02–1.17)	-	1.08 (1.01–1.16)
Type II diabetes mellitus	1.45 (0.59–3.56)	1.06 (1.00–1.13)	1.47 (0.60–3.63)	1.07 (1.00–1.13)
Metabolic syndrome	-	1.08 (1.02–1.13)	-	1.08 (1.01–1.14)
Essential (primary) hypertension	-	1.09 (1.01–1.17)	-	1.09 (1.00–1.17)
Fibrosis and cirrhosis of liver	6.82 (1.71–27.19)	1.34 (1.22–1.52)	7.85 (1.97–31.20)	1.34 (1.21–1.51)
Other disorders of liver	6.18 (2.03–18.83)	1.16 (1.02–1.32)	6.83 (2.21–21.10)	1.18 (1.06–1.31)
Renal disease	1.36 (0.39–4.75)	1.10 (1.04–1.16)	1.62 (0.46–5.70)	1.10 (1.04–1.17)
Liver cancer and cholangiocarcinoma	10.82 (6.64–17.61)	1.23 (1.13–1.35)	11.41 (6.96–18.70)	1.22 (1.12–1.32)

Men				
All-cause mortality	1.62 (1.33–1.97)	1.63 (1.28–1.94)	1.87 (1.51–2.31)	1.90 (1.52–2.25)
All endocrine diseases	1.09 (0.48–2.46)	1.05 (1.01–1.09)	1.16 (0.51–2.62)	1.04 (0.99–1.08)
All endocrine diseases (excluding T2DM)	0.25 (0.03–1.76)	1.04 (0.99–1.08)	0.26 (0.04–1.87)	1.04 (1.00–1.07)
Fibrosis and cirrhosis of liver	18.25 (6.95–47.91)	1.30 (1.19–1.47)	21.91 (8.21–58.42)	1.29 (1.19–1.45)
Diseases of gallbladder	-	1.04 (1.00–1.09)	-	1.04 (1.00–1.08)
All genitourinary diseases	1.46 (0.61–3.48)	1.04 (1.00–1.08)	1.60 (0.67–3.81)	1.03 (0.97–1.07)
All genitourinary diseases (excluding renal diseases)	1.55 (0.51–4.73)	1.03 (0.98–1.07)	1.73 (0.57–5.29)	1.03 (0.98–1.06)
Renal disease	1.30 (0.36–4.73)	1.07 (1.02–1.14)	1.37 (0.39–4.82)	1.06 (1.00–1.14)
All neoplasms	2.40 (1.81–3.18)	1.16 (1.01–1.30)	2.76 (2.06–3.70)	1.17 (1.01–1.33)
Liver cancer and cholangiocarcinoma	10.82 (7.10–16.49)	1.21 (1.13–1.35)	12.24 (7.86–19.04)	1.20 (1.12–1.34)

CHR, cumulative hazard ratio; CI, confidence interval; HCV, hepatitis C virus; T2DM, type 2 diabetes mellitus.

The symbol of "-" indicated that the model estimation did not converge.

For the risk of disease-specific mortality, hazard ratios were estimated using the Cox proportional hazards model with IPW adjustment for the propensity of HCV infection.

For the risk of disease-mediating mortality, the 25-year cumulative hazard ratios were estimated using the nonparametric causal mediation estimator with IPW adjustment.

Other disorders of the liver: excluding fibrosis and cirrhosis of the liver, and fatty liver; renal disease: nephritis, nephrotic syndrome, and nephrosis

[a] Participants were not censored when prescribing HCV drugs with IPW adjustment for the tendency of HCV treatment

[b] Participants were censored with inverse probability weighting adjustment for the tendency of HCV treatment

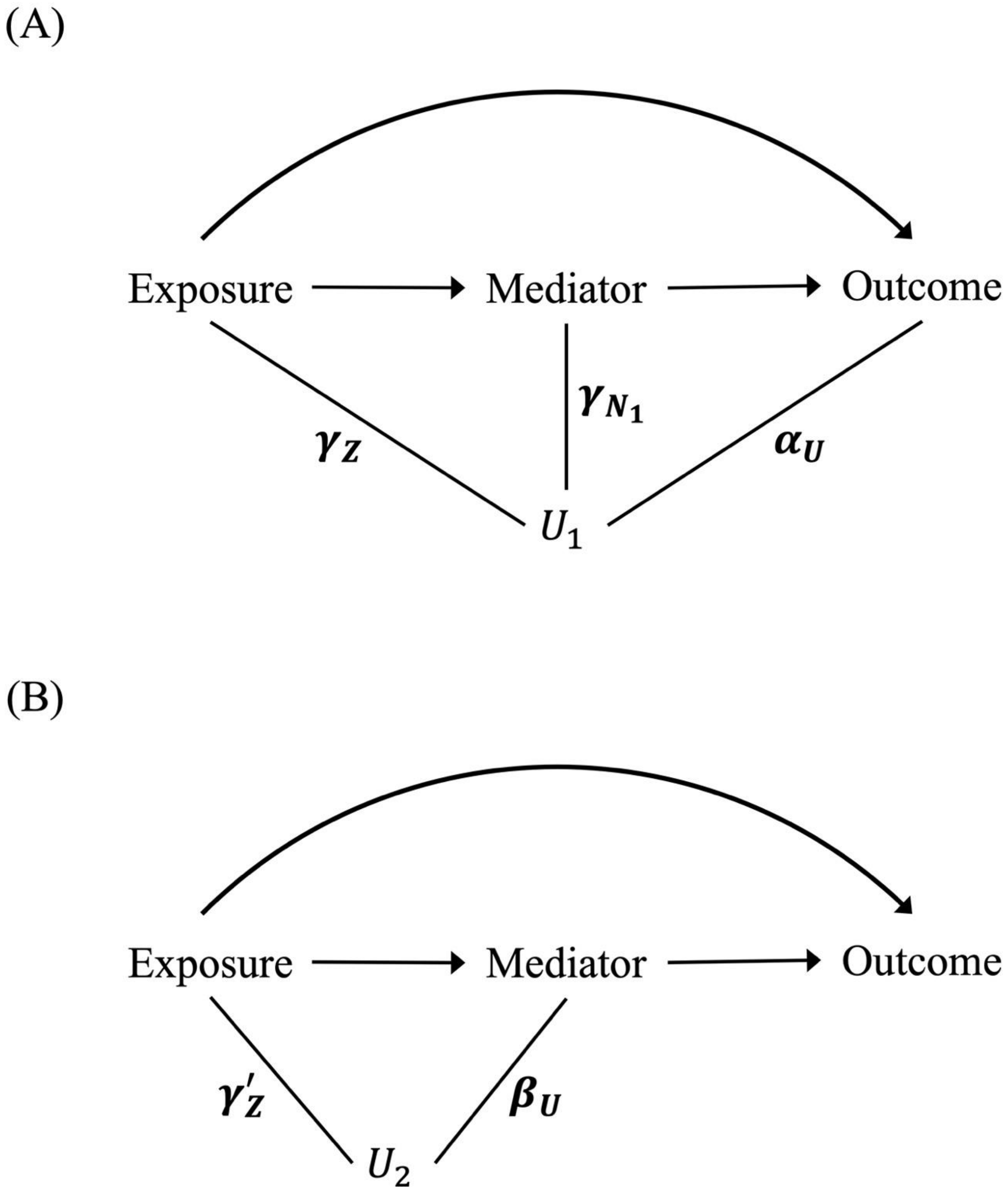

eFigure 1. Illustration of the causal structures of unmeasured confounders U_1 and U_2 in the causal diagram.

$\gamma_Z: the\ association\ of\ HCV\ and\ U_1$

$\gamma_{N_1}: the\ association\ of\ mediating\ disease\ and\ U_1$

$\alpha_U: the\ association\ of\ mortality\ and\ U_1$

$\gamma'_Z: the\ association\ of\ HCV\ and\ U_2$

$\beta_U: the\ association\ of\ mediating\ disease\ and\ U_2$

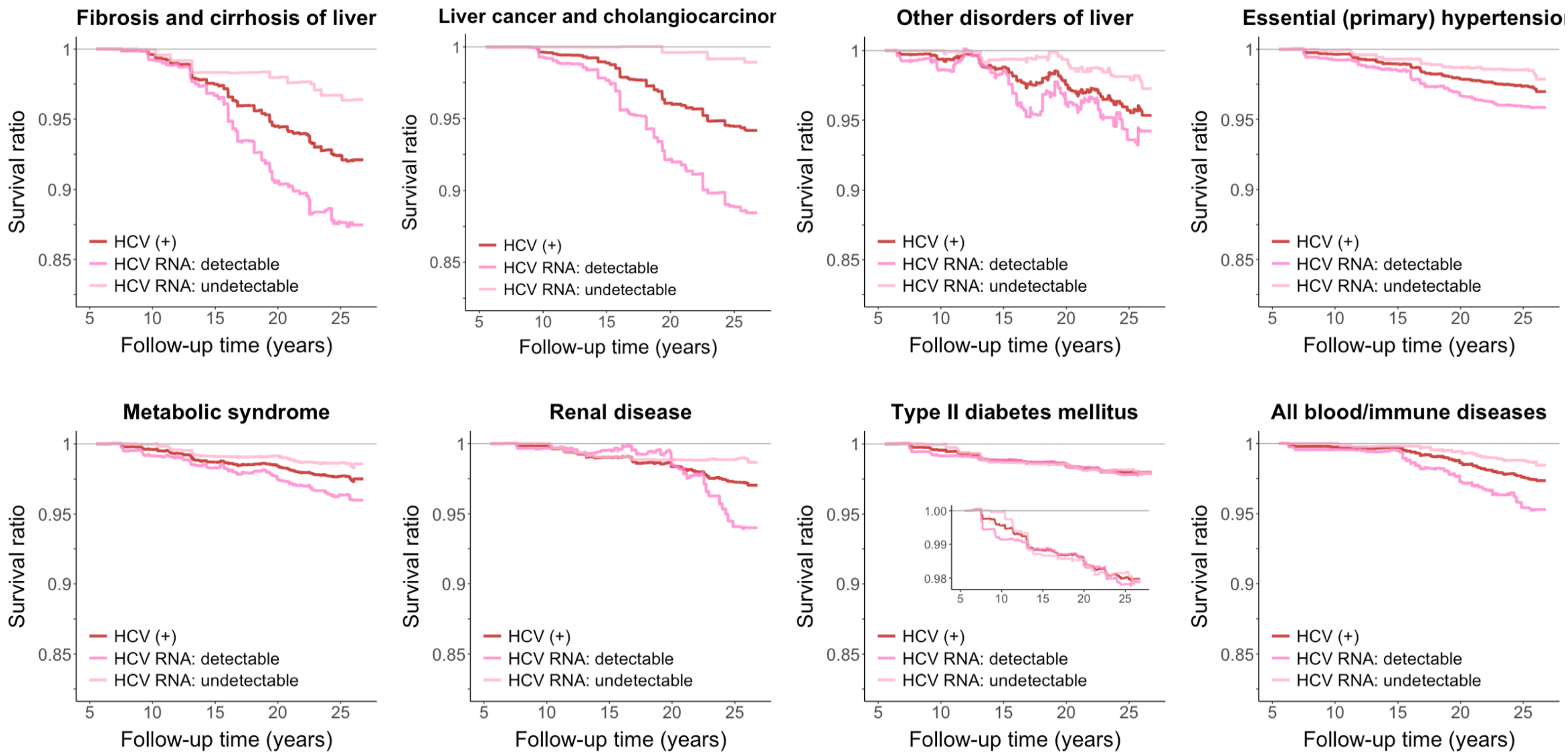

eFigure 2. Survival ratio in disease-mediating HCV-induced mortality in women stratified by HCV viral load. The survival ratio was estimated using the nonparametric causal mediation estimator with IPW adjustment for the age group (30–39 [reference], 40–49, 50–59, and 60–65 years), sex (reference: women), alcohol consumption (reference: yes), cigarette smoking (reference: yes), ALT (<15 [reference], 15–44, and ≥45 IU/L), interaction between the age group (40–49 years) and sex, interaction between the age group (40–49 years), and ALT levels (15–44 IU/L), interaction between sex and alcohol consumption, interaction between sex and ALT levels (≥45 IU/L), interaction between cigarette smoking and alcohol consumption, and interaction between cigarette smoking and ALT levels (15–44 IU/L). Other disorders of the liver: disorders of the liver excluding fibrosis and cirrhosis of the liver

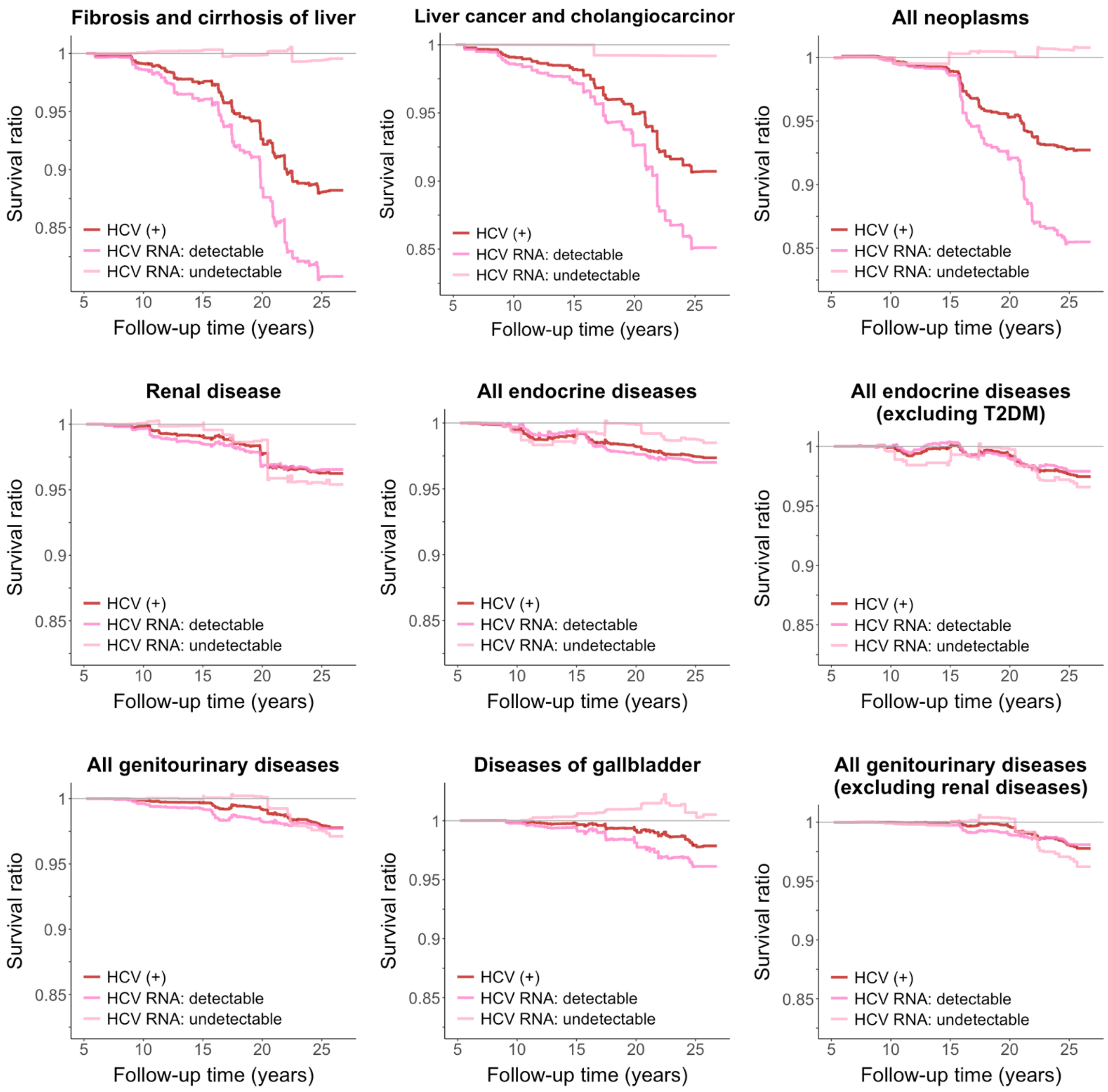

eFigure 3. Survival ratio in disease–mediating HCV-induced mortality in men stratified by HCV viral load. The survival ratio was estimated using the nonparametric causal mediation estimator with IPW adjustment for the age group (30–39 [reference], 40–49, 50–59, and 60–65 years), sex (reference: women), alcohol consumption (reference: yes), cigarette smoking (reference: yes), ALT (<15 [reference], 15–44, and ≥45 IU/L), interaction between the age group (40–49 years) and sex, interaction between the age group (40–49 years), and ALT levels (15–44 IU/L), interaction between sex and alcohol consumption, interaction between sex and ALT levels (≥45 IU/L), interaction between cigarette smoking and alcohol consumption, and interaction between cigarette smoking and ALT levels (15–44 IU/L). Other disorders of the liver: disorders of the liver excluding fibrosis and cirrhosis of the liver, and fatty liver; renal disease: nephritis, nephrotic syndrome, and nephrosis

Fibrosis and cirrhosis of liver

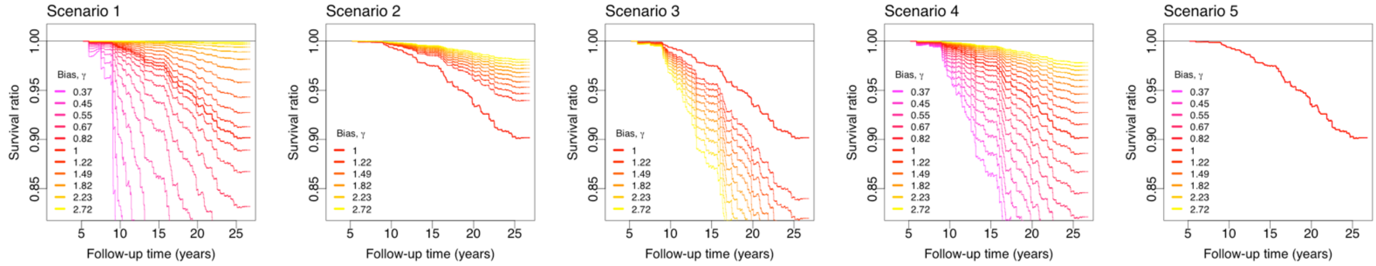

Liver cancer and cholangiocarcinoma

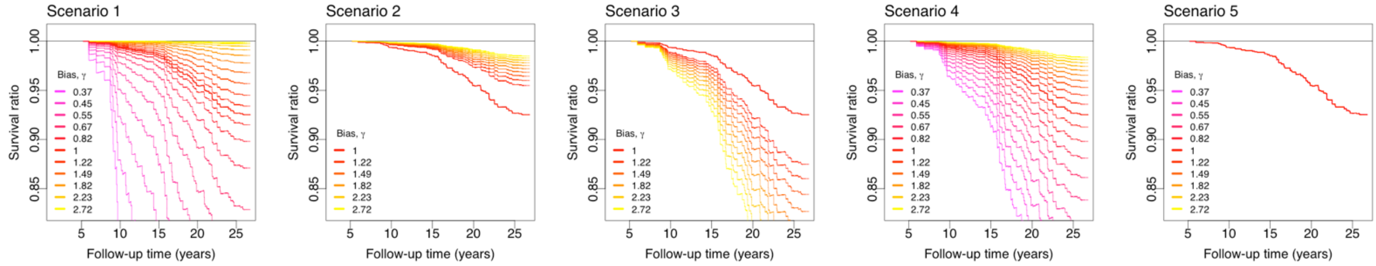

Septicemia

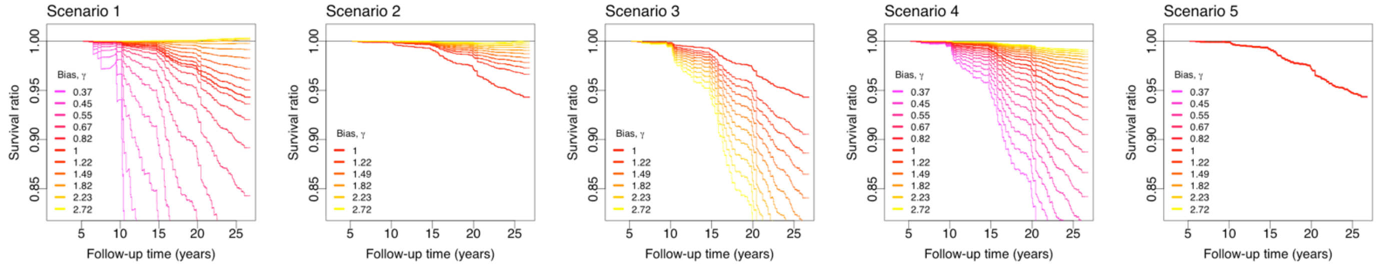

All neoplasms

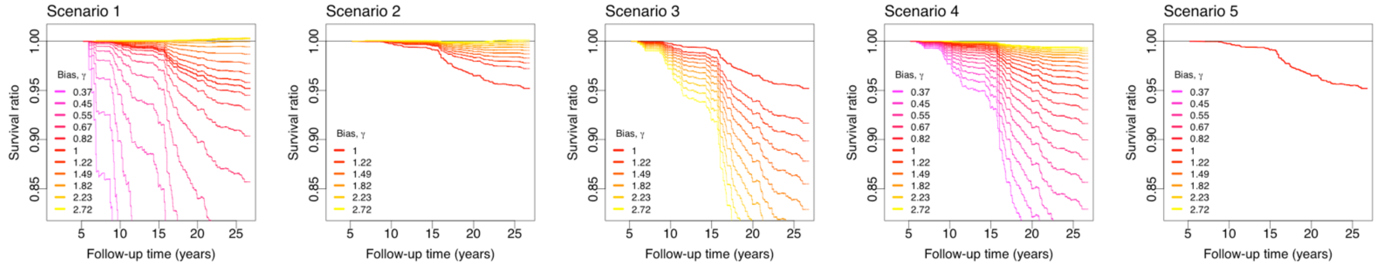

Other disorders of liver (excluding fibrosis and cirrhosis of liver and fatty liver)

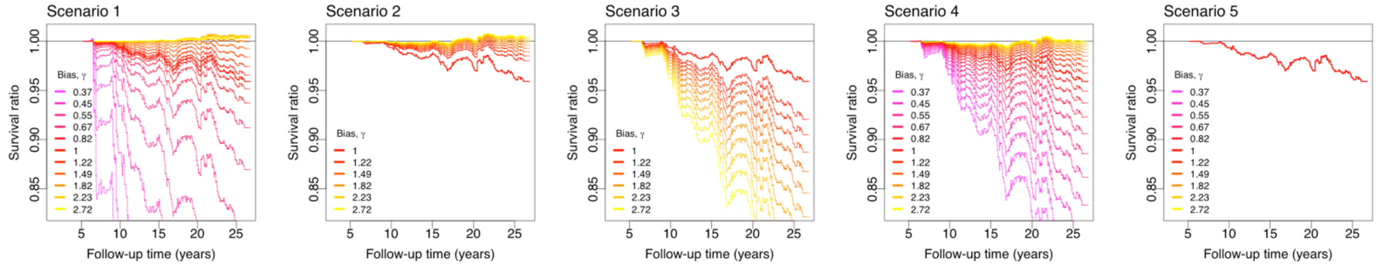

Renal disease (nephritis nephrotic syndrome and nephrosis)

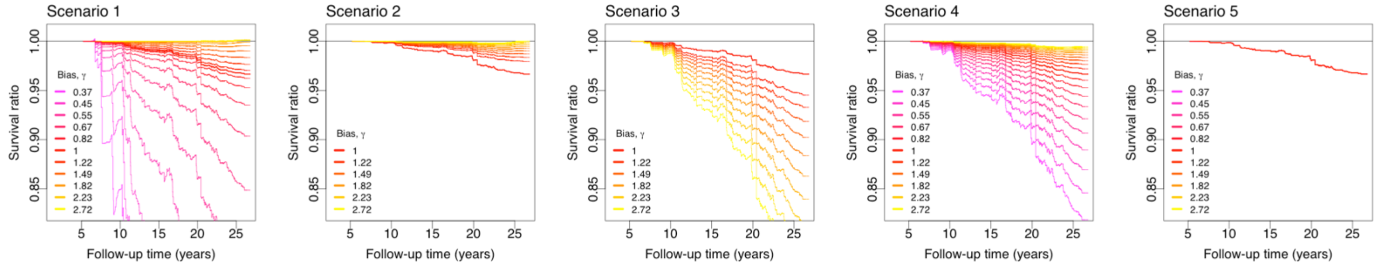

All blood/immune diseases

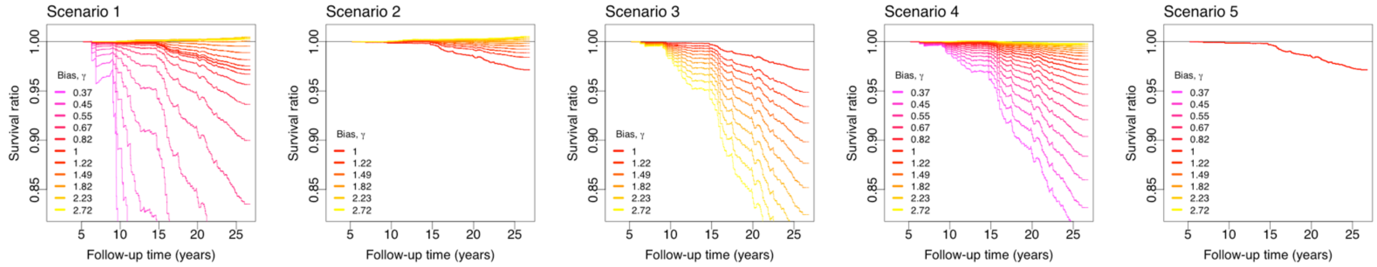

Diseases of gallbladder

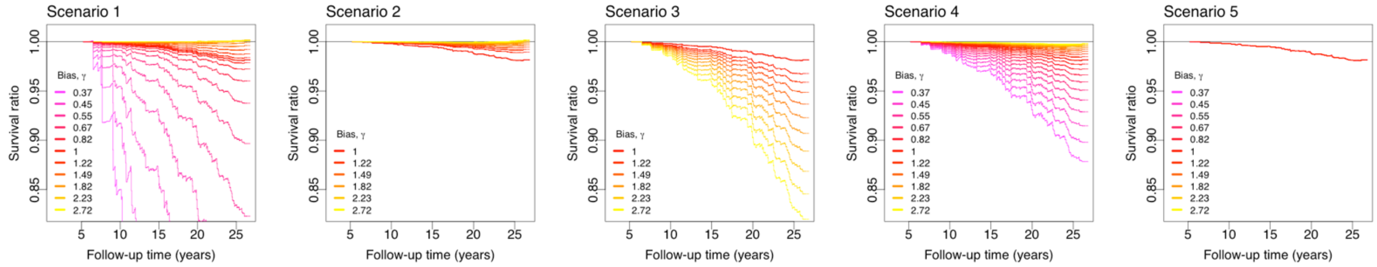

All endocrine diseases

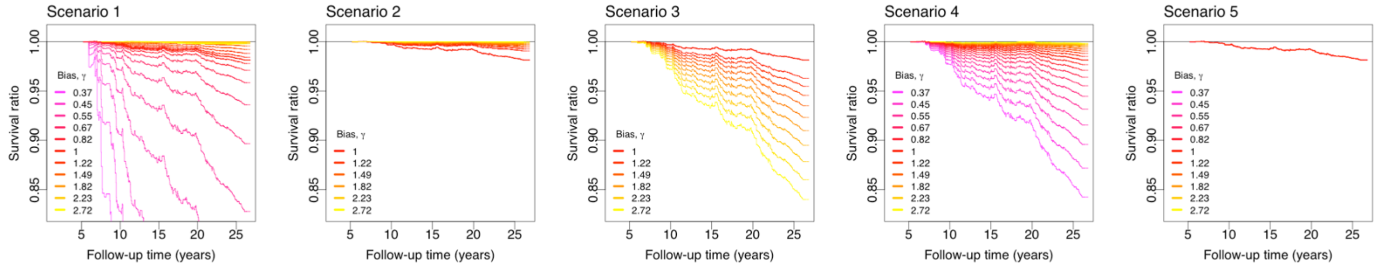

All endocrine diseases (excluding type II diabetes mellitus)

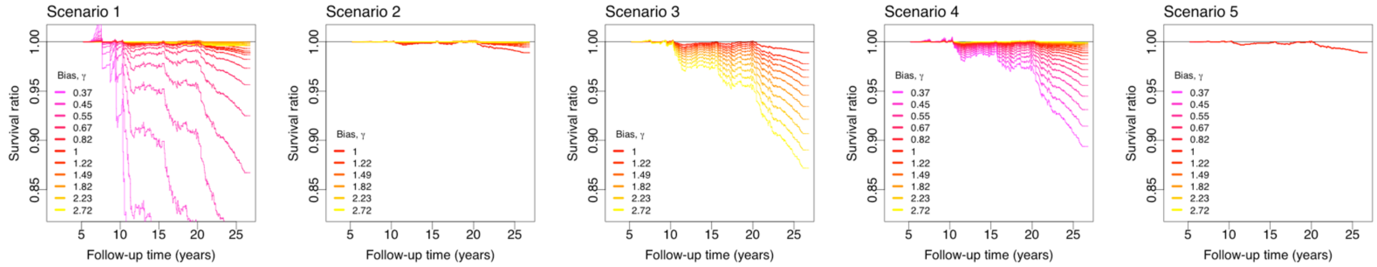

All genitourinary diseases

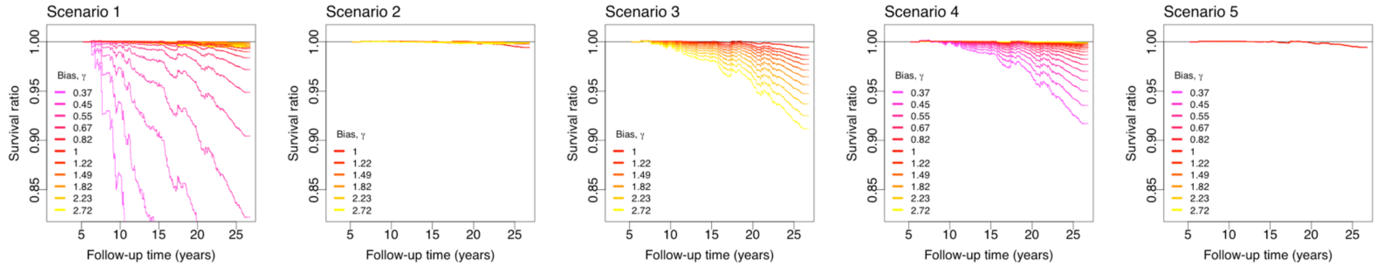

All genitourinary diseases (excluding renal diseases)

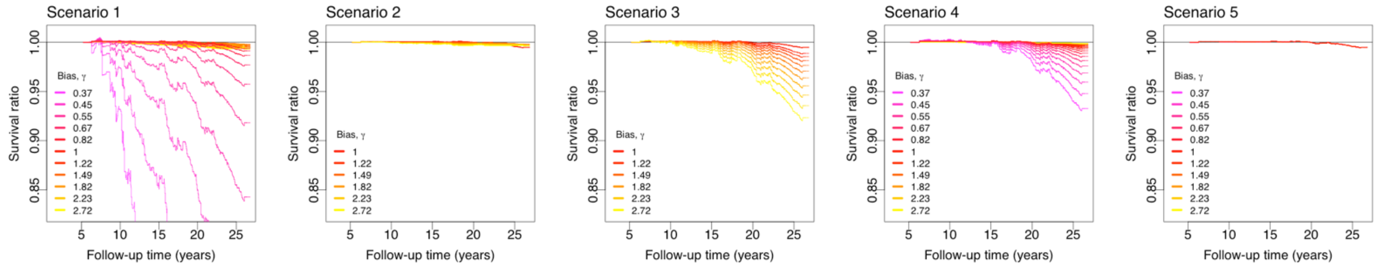

eFigure 4. Sensitivity analysis of the mediation effect of HCV-induced mortality through the 12 identified mediating diseases

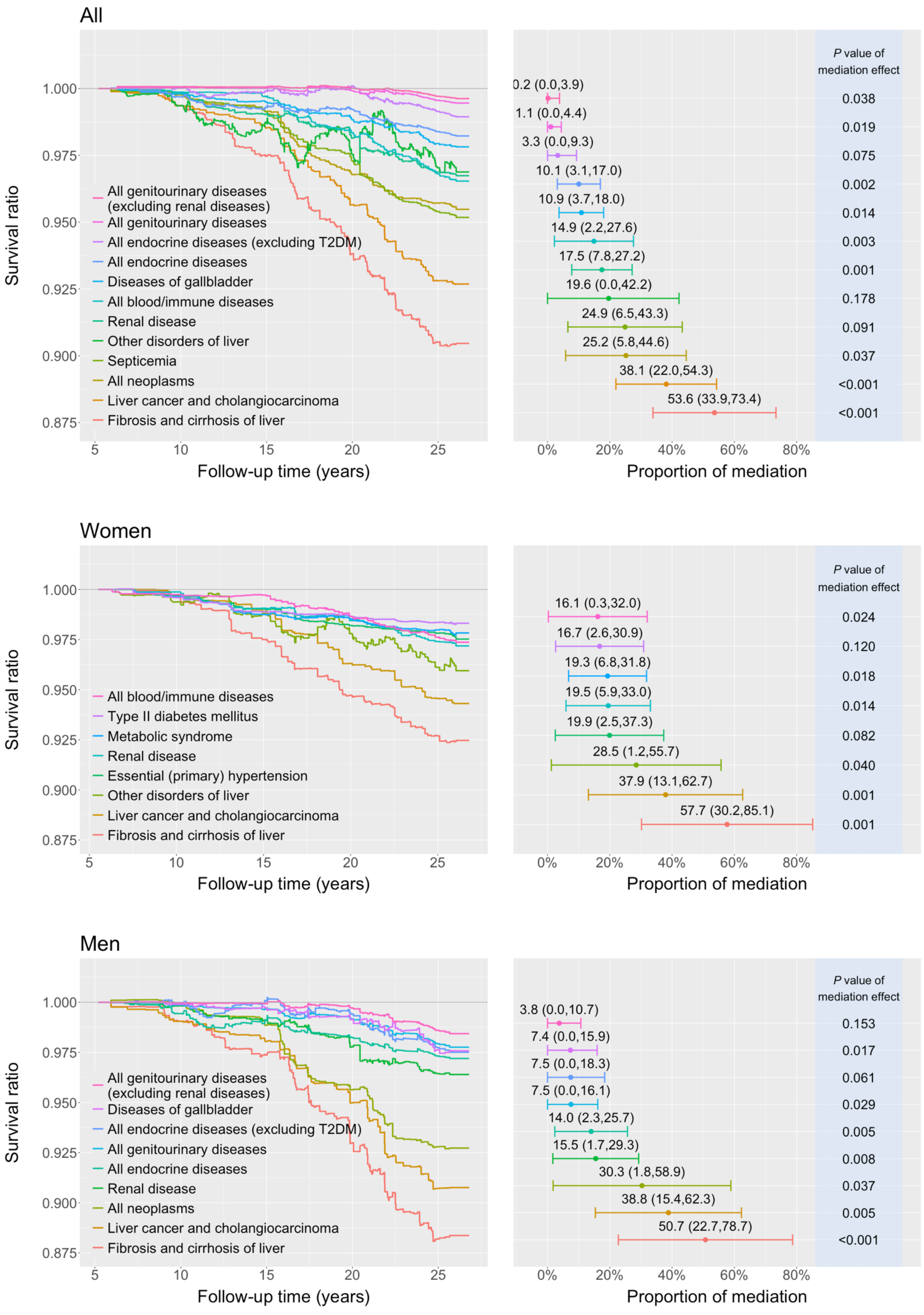
All
Survival ratio
1.000
0.975
0.950
0.925
0.900
0.875
5
10
15
20
25
Follow-up time (years)
All genitourinary diseases (excluding renal diseases)
All genitourinary diseases
All endocrine diseases (excluding T2DM)
All endocrine diseases
Diseases of gallbladder
All blood/immune diseases
Renal disease
Other disorders of liver
Septicemia
All neoplasms
Liver cancer and cholangiocarcinoma
Fibrosis and cirrhosis of liver
0.2 (0.0,3.9)
1.1 (0.0,4.4)
3.3 (0.0,9.3)
10.1 (3.1,17.0)
10.9 (3.7,18.0)
14.9 (2.2,27.6)
17.5 (7.8,27.2)
19.6 (0.0,42.2)
24.9 (6.5,43.3)
25.2 (5.8,44.6)
38.1 (22.0,54.3)
53.6 (33.9,73.4)
0%
20%
40%
60%
80%
Proportion of mediation
P value of mediation effect
0.038
0.019
0.075
0.002
0.014
0.003
0.001
0.178
0.091
0.037
<0.001
<0.001
Women
Survival ratio
1.000
0.975
0.950
0.925
0.900
0.875
5
10
15
20
25
Follow-up time (years)
All blood/immune diseases
Type II diabetes mellitus
Metabolic syndrome
Renal disease
Essential (primary) hypertension
Other disorders of liver
Liver cancer and cholangiocarcinoma
Fibrosis and cirrhosis of liver
16.1 (0.3,32.0)
16.7 (2.6,30.9)
19.3 (6.8,31.8)
19.5 (5.9,33.0)
19.9 (2.5,37.3)
28.5 (1.2,55.7)
37.9 (13.1,62.7)
57.7 (30.2,85.1)
0%
20%
40%
60%
80%
Proportion of mediation
P value of mediation effect
0.024
0.120
0.018
0.014
0.082
0.040
0.001
0.001
Men
Survival ratio
1.000
0.975
0.950
0.925
0.900
0.875
5
10
15
20
25
Follow-up time (years)
All genitourinary diseases (excluding renal diseases)
Diseases of gallbladder
All endocrine diseases (excluding T2DM)
All genitourinary diseases
All endocrine diseases
Renal disease
All neoplasms
Liver cancer and cholangiocarcinoma
Fibrosis and cirrhosis of liver
3.8 (0.0,10.7)
7.4 (0.0,15.9)
7.5 (0.0,18.3)
7.5 (0.0,16.1)
14.0 (2.3,25.7)
15.5 (1.7,29.3)
30.3 (1.8,58.9)
38.8 (15.4,62.3)
50.7 (22.7,78.7)
0%
20%
40%
60%
80%
Proportion of mediation
P value of mediation effect
0.153
0.017
0.061
0.029
0.005
0.008
0.037
0.005
<0.001

eFigure 5. HCV-induced mortality by different mediating diseases without censoring for HCV treatment.

IPW adjustment was same as that in Figure 3. The proportion of mediation was determined by computing the cumulative hazard difference and calculated as the mediation effect divided by the total effect. Confidence intervals of the mediation proportion were truncated at 0 if they were <0. Other disorders of the liver: excluding fibrosis and cirrhosis of the liver, and fatty liver; renal disease: nephritis, nephrotic syndrome, and nephrosis

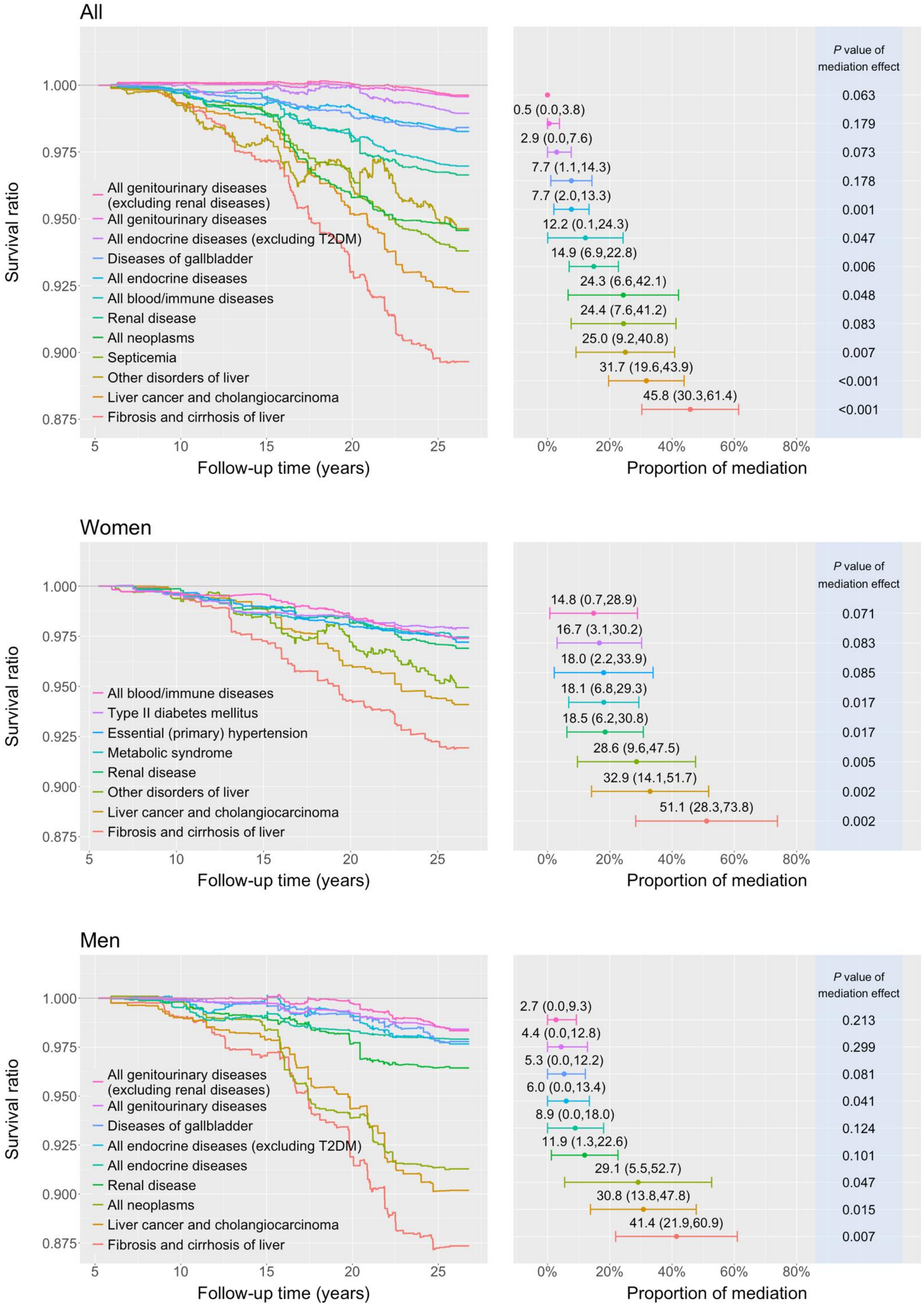
All
Survival ratio
1.000
0.975
0.950
0.925
0.900
0.875
All genitourinary diseases (excluding renal diseases)
All genitourinary diseases
All endocrine diseases (excluding T2DM)
Diseases of gallbladder
All endocrine diseases
All blood/immune diseases
Renal disease
All neoplasms
Septicemia
Other disorders of liver
Liver cancer and cholangiocarcinoma
Fibrosis and cirrhosis of liver
5
10
15
20
25
Follow-up time (years)
P value of mediation effect
0.5 (0.0,3.8)
2.9 (0.0,7.6)
7.7 (1.1,14.3)
7.7 (2.0,13.3)
12.2 (0.1,24.3)
14.9 (6.9,22.8)
24.3 (6.6,42.1)
24.4 (7.6,41.2)
25.0 (9.2,40.8)
31.7 (19.6,43.9)
45.8 (30.3,61.4)
0.063
0.179
0.073
0.178
0.001
0.047
0.006
0.048
0.083
0.007
<0.001
<0.001
0%
20%
40%
60%
80%
Proportion of mediation
Women
Survival ratio
All blood/immune diseases
Type II diabetes mellitus
Essential (primary) hypertension
Metabolic syndrome
Renal disease
Other disorders of liver
Liver cancer and cholangiocarcinoma
Fibrosis and cirrhosis of liver
Follow-up time (years)
P value of mediation effect
14.8 (0.7,28.9)
16.7 (3.1,30.2)
18.0 (2.2,33.9)
18.1 (6.8,29.3)
18.5 (6.2,30.8)
28.6 (9.6,47.5)
32.9 (14.1,51.7)
51.1 (28.3,73.8)
0.071
0.083
0.085
0.017
0.017
0.005
0.002
0.002
Proportion of mediation
Men
Survival ratio
All genitourinary diseases (excluding renal diseases)
All genitourinary diseases
Diseases of gallbladder
All endocrine diseases (excluding T2DM)
All endocrine diseases
Renal disease
All neoplasms
Liver cancer and cholangiocarcinoma
Fibrosis and cirrhosis of liver
Follow-up time (years)
P value of mediation effect
2.7 (0.0,9.3)
4.4 (0.0,12.8)
5.3 (0.0,12.2)
6.0 (0.0,13.4)
8.9 (0.0,18.0)
11.9 (1.3,22.6)
29.1 (5.5,52.7)
30.8 (13.8,47.8)
41.4 (21.9,60.9)
0.213
0.299
0.081
0.041
0.124
0.101
0.047
0.015
0.007
Proportion of mediation

eFigure 6. HCV-induced mortality by different mediating diseases with censoring for HCV treatment and inverse probability weighting adjustment. IPW adjustment was the same as that in Figure 3. The proportion of mediation was determined by computing the cumulative hazard difference and calculated as the mediation effect divided by the total effect. Confidence intervals of the mediation proportion were truncated at 0 if they were <0. Other disorders of the liver: excluding fibrosis and cirrhosis of the liver, and fatty liver; renal disease: nephritis, nephrotic syndrome, and nephrosis

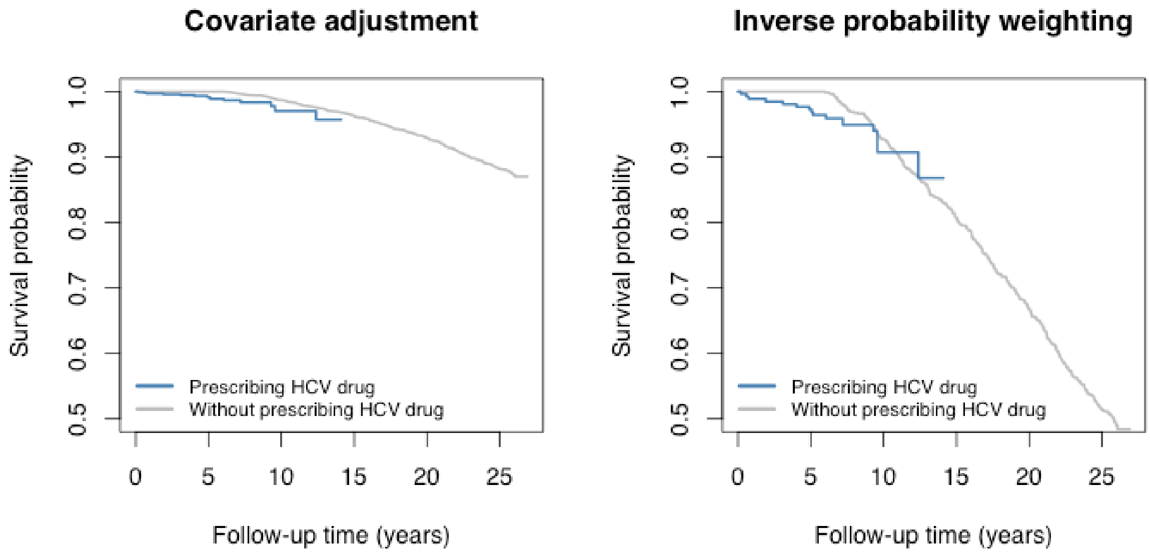

eFigure 7. Survival probability of treated and untreated HCV positive participants. The follow-up period for the treated participants started from the day of treatment. In the left panel, the survival probability was estimated using the Cox proportional hazard model with covariate adjustment for the age group (30–39 [reference], 40–49, 50–59, and 60–65 years, at the treatment if treated), sex (reference: women), alcohol consumption (reference: yes), cigarette smoking (reference: yes), ALT (<15 [reference], 15–44, and ≥45 IU/L); in the right panel, it was estimated using the Kaplan-Meier estimator with IPW adjustment for the tendency of HCV treatment.
